# Effects of the Radix Ginseng and Semen Ziziphi Spinosae drug pair on the GLU/GABA-GLN metabolic cycle and the intestinal microflora of insomniac rats based on the brain–gut axis

**DOI:** 10.3389/fphar.2022.1094507

**Published:** 2022-12-21

**Authors:** Tie Qiao, Yuan Wang, Ke Liang, Bingyuan Zheng, Jin Ma, Fangxiao Li, Chi Liu, Mingdan Zhu, Meng Song

**Affiliations:** ^1^ Liaoning Academy of Traditional Chinese Medicine Sciences, Liaoning University of Traditional Chinese Medicine, Shenyang, Liaoning, China; ^2^ Guangdong Xin-Huangpu Joint Innovation Institute of Traditional Chinese Medicine, Guangzhou, Guangdong, China; ^3^ The Second Affiliated Hospital of Liaoning University of Traditional Chinese Medicine, Shenyang, Liaoning, China

**Keywords:** Radix Ginseng–Semen Ziziphi Spinosae, drug pair, insomnia, GLU/GABA-GLN metabolic cycle, intestinal microflora

## Abstract

**Introduction:** To explore the mechanism of action of appling Radix Ginseng and Semen Ziziphi Spinosae Drug pair (R-S) in the treatment of insomnia by investigating the effect of R-S on GLU/GABA-GLN metabolic cycle and intestinal microflora of rats with insomnia.

**Methods:** Rats were intraperitoneally injected with 4-chloro-DL-phenylalanine (PCPA) to make sleep deprivation (SD) models. The rats were divided into 6 groups, with 8 rats in each group. The general status of the rats was observed and the pentobarbital sodium sleep synergy experiment was performed. The contents of GABA, GLU, GLN, GAD65, and GS in hippocampus of rats were determined by ELISA. The expressions of GABAARα1mRNA, mGluR5mRNA, NR1mRNA and GluR1mRNA in rats’ hippocampal tissue were determined by Realtime PCR. 16SrRNA gene sequencing was used to analyze the intestinal microflora of insomnia rats.

**Results:** In PCPA-induced insomnia rats, the state of insomnia was relieved, the sleep rate was improved, the duration of sleep latency was shortened and the sleep duration was prolonged in each dose group of R-S (*p* < 0.05, *p* < 0.01) compared with the model group. The contents of GABA, GLN, GAD65 and GS were increased (*p* < 0.05, *p* < 0.01) while GLU content was decreased (*p* < 0.01) in both medium and high dose groups, especially in the high dose group. The expression of GABAARα1mRNA was increased (*p* < 0.01), and the expressions of mGluR5mRNA, NR1mRNA and GluR1mRNA were decreased (*p* < 0.01) in hippocampal tissue of rats in R-S groups, especially in the high dose group. At the same time, the various dose groups of R-S could improve the species diversity, microflora abundance of insomnia rats and regulate the KEGG metabolic pathway related to sleep.

**Discussion:** R-S can improve the sleep of PCPA-induced insomnia rats by regulating GLU/GABA-GLN metabolic cycle and intestinal microflora, which provides experimental basis for appling R-S in the treatment of insomnia.

## 1 Introduction

Insomnia is a condition where patients have difficulty falling asleep, easily wake up, or cannot fall asleep after waking up. Traditional Chinese medicine (TCM) believes that the pathogenesis of insomnia is nothing more than the uneasiness aroused by a disorder of the body’s function. Western medicine believes that the etiology of insomnia is complex, including physiology, psychology, age, and society; its pathogenesis is mainly believed to be related to the abnormal gamma-aminobutyric neuron system, central neurotransmitter disorder, and hypothalamic–pituitary–adrenal axis dysfunction (Chow and Cao, 2016; Vgontzas et al., 2020; Yang X. et al., 2021). Clinical drugs commonly used by Western medicine in the treatment of insomnia are divided into benzodiazepines (BZD) and non-BZD, with BZD being the first choice. However, both often induce drowsiness, dependence, withdrawal symptoms, and other side effects (Hua et al., 2021). TCM has a history of thousands of years in the treatment of insomnia and enjoys good clinical effects. It has a lower addiction tendency and fewer side effects. For instance, the Radix Ginseng and Semen Ziziphi Spinosae drug pair (R-S) is often used in the clinical treatment of insomnia. They are often combined in formulas such as Returning to Spleen Decoction (Gui Pi Tang), Heart-Nourishing Decoction (Yang Xin Tang), Celestial Emperor Heart-Tonifying Pill (Tian Wang Bu Xin Dan), and their compatibility can enhance the tranquilizing effect on the mind.

The brain–gut axis is a two-way information exchange system between the brain and intestine which is connected by the neuroendocrine, immune, and metabolic pathways. Intestinal microflora, an important link in the brain–gut axis, plays an important role in regulating digestion and absorption of the host, intestinal endocrine function, immune function, toxin clearance, and metabolism. Steady-state intestinal microflora maintains the integrity of the intestinal epithelial barrier and assists the host in resisting pathogen invasion. If the steady state between the intestinal microflora and the host is destroyed, then this flora will undermine the health of the host in, for example, the brain–gut axis, short-chain fatty acids, endotoxemia, energy absorption, choline, and the bile acid metabolism (Janssen and Kersten, 2015). Intestinal microflora plays an important role in the neuroendocrine pathway. By stimulating the afferent neurons of the intestinal nervous system, the intestinal microflora generates synaptic connections between the intestinal and the vagus nerves, forming information transmission between the intestinal microflora and the brain (Li et al., 2018). Studies have shown that the disorder of intestinal microflora may be the mechanism leading to insomnia (Cryan John et al., 2019). For example, in the case of short-time sleep maintenance and fragmented sleep, the body breaks the balanced state of intestinal microflora by activating the HPA axis (Matenchuk Brittany et al., 2020), leading to the decline of beneficial bacteria and changing their metabolic function and level (Bowers et al., 2020).

Radix Ginseng is the dry root and rhizome of *Panax ginseng* C. A. Meyer. It has the effect of nourishing the heart and spleen, calming the mind, and intensifying intelligence. It is used for symptoms such as deficiency of the spleen and eating loss, palpitations, and insomnia. Semen Ziziphi Spinosae is the dry and mature seed of *Ziziphus jujube* (*Z. jujuba* Mill.var.*spinosa* (Bunge) HuexH.F.Chou), which has the effect of nourishing the heart, tonifying the liver, and calming the mind. It is suitable for symptoms such as irritation due to fatigue and insomnia, palpitations, and dreamfulness. According to the literature, ginsenoside Rg5/Rk1 can improve the GABA/GLU ratio and the expressions of the GABAA and GABAB receptors, thus improving sleep quality (Shao et al., 2020). Experimental studies have found that Semen Ziziphi Spinosae affects insomnia mainly by changing the contents of GABA and 5-HT, thus having a sedative and hypnotic effect (Yan J. et al., 2019). Studies have found that total saponins of Semen Ziziphi Spinosae can restore the intestinal microflora of insomniac rats to maintain the homeostatic flora (Cui, 2020). It was found that potential probiotics were up-regulated and the harmful bacterium TM7 was inhibited in the intestinal microflora of rats which were given ginseng extract (100 mg/kg) by long-term instillation (Sun et al., 2018). Li et al. (2020) found that R-S extracts (mass ratio 3:5) could significantly improve the relative abundance of Bacteroides and Firmicutes in insomniac rats, while the relative abundance of Actinobacteria decreased. Additionally, the extracts could significantly increase the relative abundance of beneficial bacteria in insomniac rats and reduce the relative abundance of *Bacillus coli*, Enterococcus, and other harmful bacteria.

It can be seen from the above that R-S can both relieve insomnia by affecting the level of neurotransmitters and expressions of receptors in the brain, and also regulate intestinal microflora. However, there has been no research on the compatibility mechanism of the two in the treatment of insomnia. They two are often used together in clinical practice to nourish the spleen and calm the mind. Therefore, in this study, intraperitoneal PCPA injection was used to establish a rat model of insomnia. Based on the pharmacodynamics test of R-S, the mechanism of R-S in the treatment of insomnia was explored by studying the effect of R-S on the GLU/GABA-GLN metabolic cycle and the intestinal microflora of insomniac rats.

## 2 Materials and methods

### 2.1 Chemicals and reagents

Radix Ginseng and Semen Ziziphi Spinosae were sourced from Bozhou Sanfeng TCM decoction pieces Co., Ltd. (Hebei, China); both were authenticated by Professor Yanjun Zhai from Liaoning University of Traditional Chinese Medicine. 4-Chloro-DL-phenylalanine (PCPA) was purchased from Sigma-Aldrich Co., Ltd. (United States). GABA, GLU, GLN, GS, and GAD65 ELISA Kits were provided by AMEKO (Shanghai, China). Prime® Script RT Reagent Kit with gDNA Eraser, SYBR^®^Premix Ex Taq TMII (TliRNaseH Plus) were provided by Takara (Japan). Primer sequences were synthesized by Shanghai Sangong Bioengineering Co., Ltd. (Shanghai, China). Quant-iT PicoGreen dsDNA Assay Kit and TruSeq Nano DNA LT Library Prep Kit were purchased from Illumina (United States).

Following the Chinese pharmacopoeia, aqueous extraction was selected. R-S were soaked for 30 min in a 1:1 ratio of high, middle, and low dosage, respectively, and then boiled in 500 ml water for 30 min. The two decoctions were then mixed and concentrated into a crude drug containing R-S 0.216 g/ml, 0.163 g/ml, and 0.108 g/ml, respectively. After cold storage, the supernatant was centrifuged (15 min, 5,000 r/min). It was put into the material tank as the ultrafiltration material liquid, and the ultrafiltration pump was opened to extract and filter out a liquid of traditional Chinese medicine.

### 2.2 Animals and treatment

A total of 48 SPF male SD rats (180 ± 20 g) were purchased from Liaoning Changsheng Biotechnology Co., Ltd. (SCXK 2020-0001, Liaoning, China). These were randomly divided into six groups, including a control group (K), model group (M), diazepam group (DX), and G-Z groups with three R-S dosages (2.16 g/kg, 1.62 g/kg, 1.08 g/kg): a high-dose (G), medium-dose (Z), and low-dose (D) group (n = 8). All rats were fed in the Experimental Animal Center of Liaoning University of TCM (humidity 50%–65% at 18°C–24°C), with a 12-h light/dark cycle. Each group was allowed to eat and drink uncontrollably every day, and their bedding was changed every 2 days.

After a week of adaptive feeding, all rats were intraperitoneally injected with PCPA suspension (0.04 g/ml/d) according to method, with minor modifications (Lv et al., 2021; Guo et al., 2021) for 3 consecutive days; the same volume of saline was injected into the control group. Symptoms such as loss of circadian rhythm, shaggy hair or hair removal, reduced diet, and strong stress reaction were evidence of the model’s success. From the 4th day on, the dose concentrations of the rats in each group were administered thus: high-dose R-S group 2.16 g/kg, medium-dose R-S group 1.62 g/kg, low-dose R-S group 1.08 g/kg, and diazepam group 0.001 g/ml. The rats of each group were intragastrically perfused with 2 ml every day. The model and control groups were given 2 ml distilled water every day for 15 consecutive days.

### 2.3 Sample collection

After an intragastric intervention, the rats were placed on the ultra-clean workbench and their lower abdomens were stimulated with a creep to defecate three to four particles. The feces were placed in a frozen tube and quickly transferred to a refrigerator at –80°C for storage. After the feces were collected, the rats were injected with 0.3 ml/100 g chloral hydrate to put them into a state of deep anesthesia. Their brains were taken out on the super-clean workbench and placed in the ice box. Their hippocampi were quickly separated according to the brain localization map, placed in EP tubes, preserved in liquid nitrogen, and quickly transferred to the refrigerator at −80°C.

### 2.4 Observation of rats’ state

The rats’ mental state (malaise or activity), diet (more, less, or normal), body weight (slow or normal weight gain), hair state (smooth or shaggy), and stress reaction (severe uncontrollable or controllable) were observed.

### 2.5 Pentobarbital sodium sleep synergistic experiment

Throughout the pre-experiment, the suprathreshold dose of pentobarbital sodium was 50 mg/kg and the subthreshold dose was 40 mg/kg. Six rats in each group were selected for the experiment. 1) Subthreshold dose experiment: pentobarbital sodium of 40 mg/kg was injected intraperitoneally in each group after intragastric administration, and the number of rats falling asleep in each group was observed and recorded. 2) Suprathreshold dose experiment: pentobarbital sodium of 50 mg/kg was injected after intragastric administration; the injection time, the disappearance time of righting reflex (with 1 min as the standard), and the sleep duration (recovery of righting reflex as the standard) were observed and recorded for each group.

### 2.6 Analysis of the contents of GABA, GLU, GLN, GAD65, and GS in the hippocampi of the rats

Sodium chloride solution was added to 0.1 g hippocampal tissue and homogenized in a high-flux tissue grinder; 10% hippocampal homogenate was prepared. This was put into a centrifuge and centrifuged (at 4°C, 3000 r/min for 20 min) to obtain the supernatant. After treatment according to the instructions of the ELISA kit, the GABA, GLU, GLN, GS, and GAD65 content in the hippocampal tissue of rats was detected by an enzyme labeling instrument.

### 2.7 Expressions of GABAAR α 1mRNA, mGluR5mRNA, NR1mRNA, and GluR1mRNA in rat hippocampi

A quantity of 0.1 g hippocampal tissue was quickly ground in liquid nitrogen, and mRNA was extracted from the tissue using Trizol, chloroform, isopropanol, and DEPC water. The purity and concentration of RNA were then determined. We configured 10 μL of DNA removal reaction solution, took a 1 μL RNA sample, and added the reaction solution to 10 μL, which was put into a PCR instrument to react (at 42°C for 2 min). The relative expressions of GABAAR α 1mRNA, mGluR5mRNA, NR1mRNA, and GluR1mRNA were calculated according to the 2-deltact (Livak) method. In the primer sequence, the primer design used Prime-BLAST (Table 1).

**TABLE 1:** Table of primer sequences.

Gene name	Reference sequence	Primer sequences (5′-3′)		Product size (bp)
GABAAα1mRNA	NM_183,326	F	CGT​GGT​TCC​AGA​AAA​GCC​AA	79
		R	GCT​GGT​TGC​TGT​AGG​AGC​AT	
mGluR5mRNA	NM_017012	F	TGG​GGA​AAC​CCT​AAG​CTC​CA	90
		R	GAC​AGT​CGC​TGC​CAC​AAA​TG	
GluR1mRNA	NM_031608	F	GTC​GAA​GCG​GAT​GAA​GGG​TT	70
		R	GGT​CGA​TGT​CCG​TAT​GGC​TT	
		R	CTC​GGG​AAG​GCA​CAG​CAA​TA	
β-actin	NM_031144	F	CGC​GAG​TAC​AAC​CTT​CTT​GC	70
		R	CGT​CAT​CCA​TGG​CGA​ACT​GG	

### 2.8 Analysis of intestinal microflora in rats with insomnia

The intestinal microflora of insomniac rats was analyzed by 16SrRNA gene sequencing technology. The above six groups of samples were delivered to Shanghai Paisenol Biotechnology Co., Ltd., and 16SrRNA gene sequencing technology was used to analyze the intestinal microflora. 1) The total DNA of the sample was extracted by a fecal DNA extraction kit and was quantified by NanoDrop. The extraction quality of DNA was detected by 1.2% agarose gel electrophoresis. 2) The corresponding primers were designed according to the conserved region of the sequence, and the variable region of the rRNA gene was amplified by PCR. 3) The PCR amplification products were purified by magnetic beads and quantified by fluorescence. 4) The TruSeq Nano DNA LT Library Prep Kit of Illumina was used to prepare the sequencing library. 5) MSeq sequencer (Illumina) was used for double-terminal sequencing; the corresponding reagent was MiSeq reagent Kit V3 (600cycles). The optimal sequencing length of the target fragment was 200-450bp. 6) Using QIIME software, high-quality sequences were obtained by quality control, denoising, splicing, and de-chimerism, and then classified according to 97% sequence similarity and operational taxonomic unit (OTU). 7) The most abundant sequence in each OTU was compared with the template sequence in the Greengenes database (Release13.8), obtaining the microflora of all OTUs at the classification levels of microbiology—including phylum, family, and genus. The fecal intestinal microflora was thus analyzed by species composition analysis, diversity analysis, and KEGG metabolic pathway prediction analysis.

## 3 Results

### 3.1 Effects of R-S on the general state of insomniac rats

**FIGURE 1:**
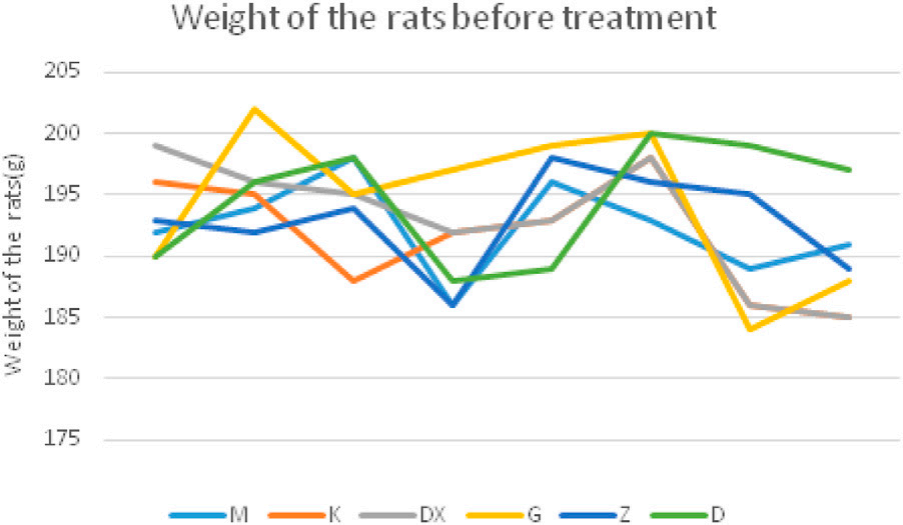
Body weight curve in different groups of rats before treatment.

**FIGURE 2:**
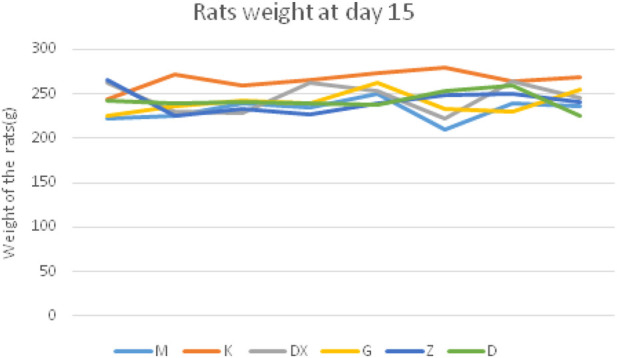
Body weight curve in different groups of rats at the 15th day.

The rats in the control group were in a good mental state, having eaten an acceptable diet, gradually increased body weight, had smooth hair, and a slightly controllable stress response. Compared with the control group, rats in the model group were in low spirits, having eaten less food, gaining weight slowly, having dull and shaggy hair, and a strong stress response. Compared with the model group, the mental state, diet, weight, and other general states of the rats in the R-S administration groups were significantly improved, while the rats in the diazepam group were in a state of not resisting handling and being lethargic. The body weight curve of rats is described in Figure 1 and Figure 2.

### 3.2 Effects on the number of rats falling asleep induced by the subthreshold dose of pentobarbital sodium

**FIGURE 3:**
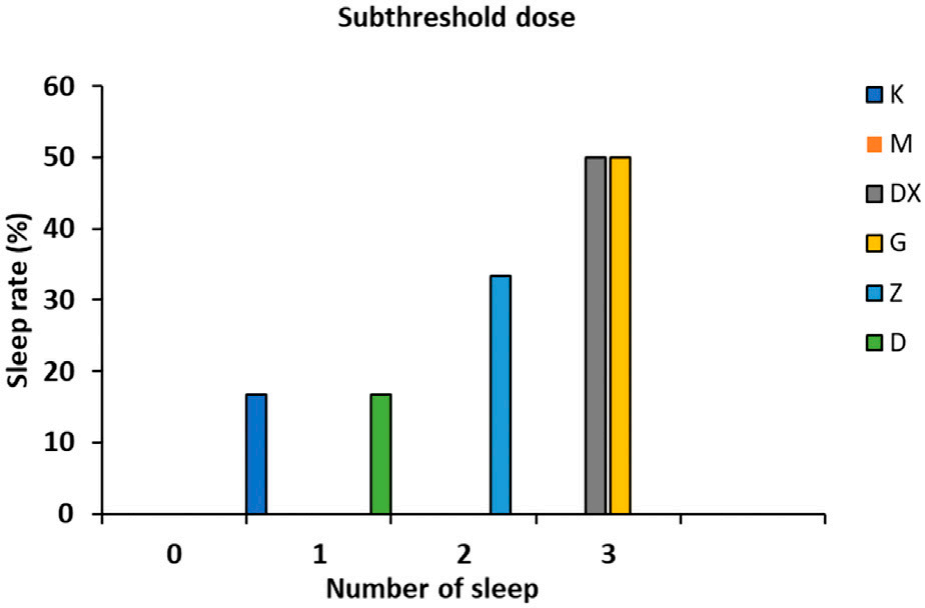
Effects of R-S on the subthreshold dose of pentobarbital sodium synergistic sleep experiment in rats with insomnia.

As shown in Figure 3, no sleeping rats appeared in the model group. There were three sleeping rats in the diazepam group and the positive rate was up to 50.00%. There were three, two, and one sleeping rat in the high-, medium-, and low-dose groups, respectively, and the positive rates were 50.00%, 33.33%, and 16.66%, respectively, which all increased significantly.

### 3.3 Effects on sleep latency and sleep duration induced by the suprathreshold dose of pentobarbital sodium in rats

**FIGURE 4:**
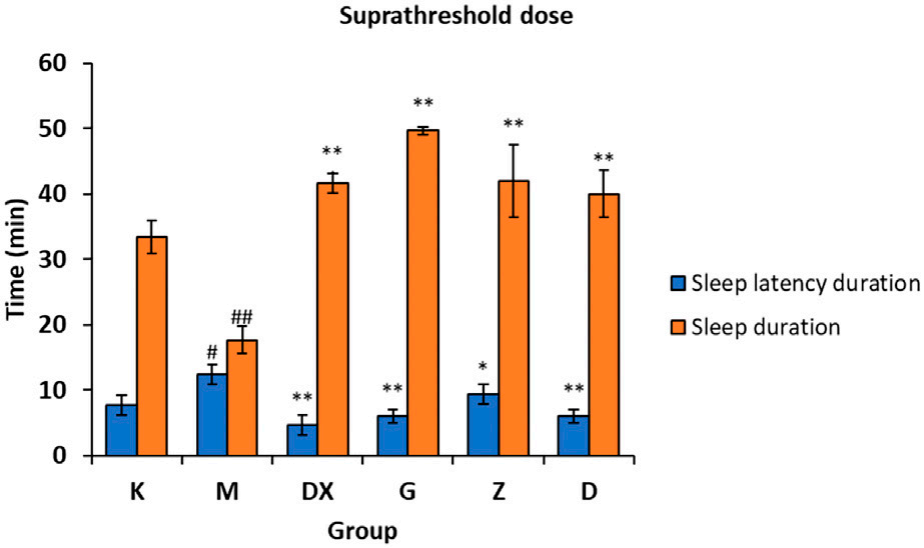
Effects of R-S on the suprathreshold dose of pentobarbital sodium synergistic sleep experiment in rats with insomnia (^#^ *p* < 0.05, ^##^ *p* < 0.01 vs. K;*p < 0.05, **p < 0.01 vs. M.).

As shown in Figure 4, compared with the control group, the sleep latency of the model group was significantly prolonged (*p* < 0.05) and the sleep duration was significantly shortened (*p* < 0.01) after administration. Compared with the model group, the sleep latency of the diazepam group and the high, medium, and low-dose R-S groups was shortened (*p* < 0.05, *p* < 0.01), and the sleep duration was significantly prolonged (*p* < 0.01).

### 3.4 Effects of R-S on the contents of GABA, GLU, GLN, GAD65, and GS in the hippocampi of insomniac rats

**FIGURE 5:**
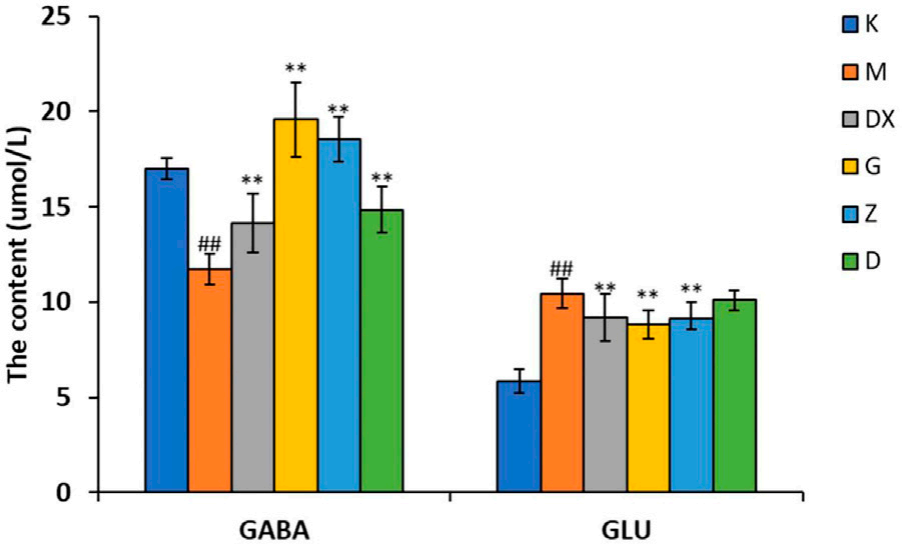
Effects of R-S on GABA and GLU content in rats with insomnia.

**FIGURE 6:**
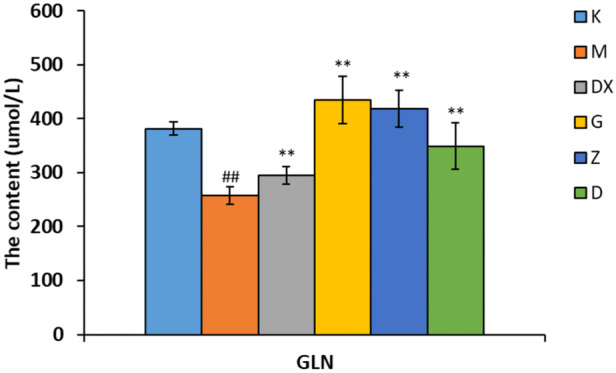
Effect of R-S on GLN content in rats with insomnia.

**FIGURE 7:**
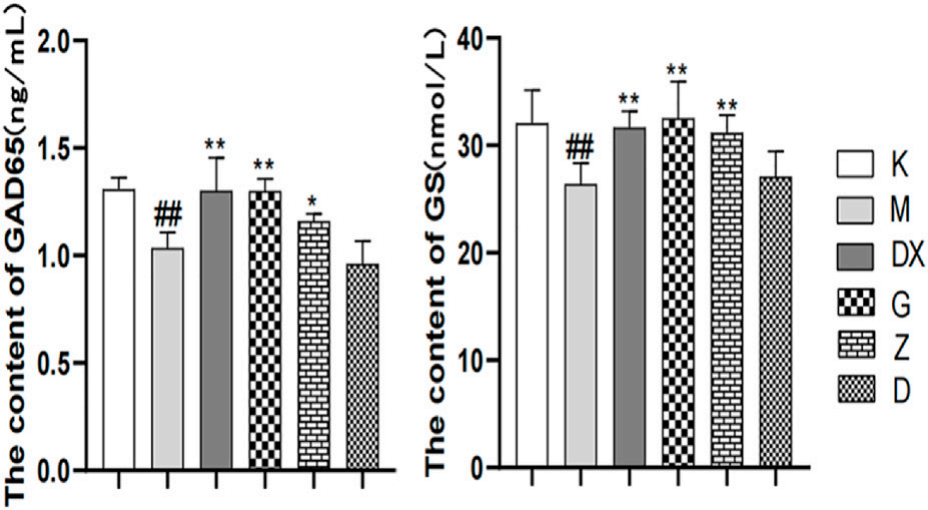
Effects of R-S on GAD65 and GS content in rats with insomnia.

As shown in Figures 5–7, compared with the control group, the contents of GABA and GLN in the hippocampi of the rat model group were significantly decreased (*p* < 0.01). Compared with the model group, the contents of GABA and GLN in the hippocampi of rats of the diazepam and the high-, medium- and low-dose R-S groups showed significant increase (*p* < 0.01). Compared with the control group, the GLU content of hippocampi in the model group significantly increased (*p* < 0.01). The GLU content of hippocampi in diazepam and the medium and high-dose R-S groups significantly decreased compared with the model group (*p* < 0.01). The low-dose R-S group had no significant difference to the model group. Compared with the control group, the contents of GAD65 and GS in the hippocampi of the model group were significantly decreased (*p* < 0.01), and the contents of GAD65 and GS in the hippocampi of the diazepam and the medium- and high-dose R-S groups were significantly increased compared with the model group (*p* < 0.01, *p* < 0.05). However, there was no significant difference between the low-dose group and the model group.

Insomniac rats were as described in the experiment. They were given medication or distilled water orally every day from the first day of insomnia. On the 16th day of insomnia, the expressions of GABA and GLU in the hippocampi of rats were detected by ELISA (*n* = 8 for each group). The results are expressed as the mean ± SD, ^#^ *p* < 0.05, ^##^ *p* < 0.01 vs. K; ^*^ *p* < 0.05, ^**^ *p* < 0.01 vs. M.

Insomniac rats were as described in the experiment. They were given medication or distilled water orally every day from the first day of insomnia. On the 16th day of insomnia, the expressions of GLN in the hippocampi of rats were detected by ELISA (*n* = 8 for each group). The results are expressed as the mean ± SD, ^#^ *p* < 0.05, ^##^ *p* < 0.01 vs. K; ^*^ *p* < 0.05, ^**^ *p* < 0.01 vs. M.

The effects of Radix Ginseng–Semen Ziziphi Spinosae on GAD65 and GS contents of the hippocampi of insomniac rats were regulated, as the insomniac rats described in the experiment were given medication or distilled water orally every day from the first day of insomnia. On the 16th day of insomnia, the expressions of GAD65 and GS in the hippocampi of rats were detected by ELISA (*n* = 8 for each group). ^#^ *p* < 0.05, ^##^ *p* < 0.01 vs. K; ^*^ *p* < 0.05, ^**^ *p* < 0.01 vs. M.

### 3.5 Effects of R-S on the expressions of GABAARα1mRNA, mGluR-5mRNA, NMDA-NR1mRNA, and GluR1mRNA in the hippocampi of insomniac rats

**FIGURE 8:**
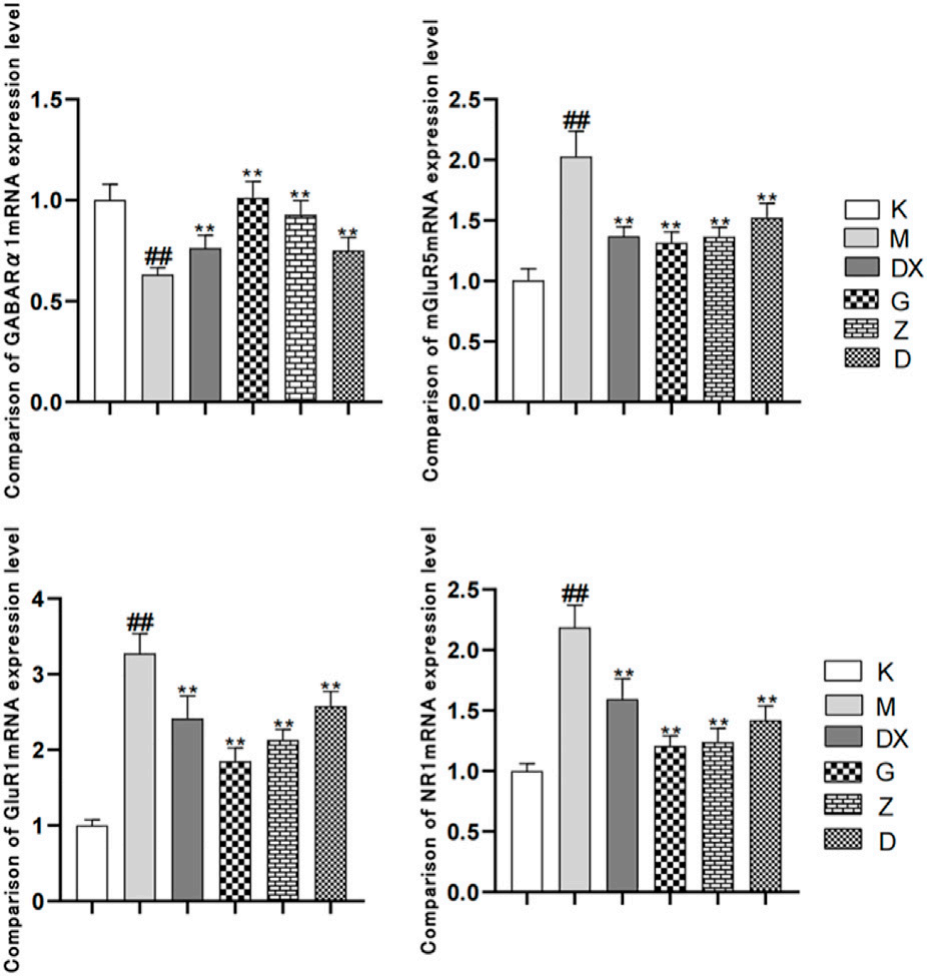
Effects of R-S on the expression of mGluR5mRNA, NR1mRNA, and GluR1mRNA in rats with insomnia.

As shown in Figure 8, compared with the control group, the GABAARα1mRNA level in the hippocampi of the model group was significantly decreased (*p* < 0.01). Compared with the model group, the levels of GABAARα1mRNA in the hippocampi of the diazepam and the high-, medium- and low-dose R-S groups were significantly increased, and the difference was statistically significant (*p* < 0.01). Compared with the control group, the expressions of mGluR5mRNA, NR1mRNA, and GluR1mRNA in the hippocampi of the model group were significantly increased (*p* < 0.01). Compared with the model group, the expressions of mGluR5mRNA, NR1mRNA, and GluR1mRNA in the diazepam, high-, medium- and low-dose R-S groups were significantly decreased and the difference was statistically significant (*p* < 0.01).

**FIGURE 9:**
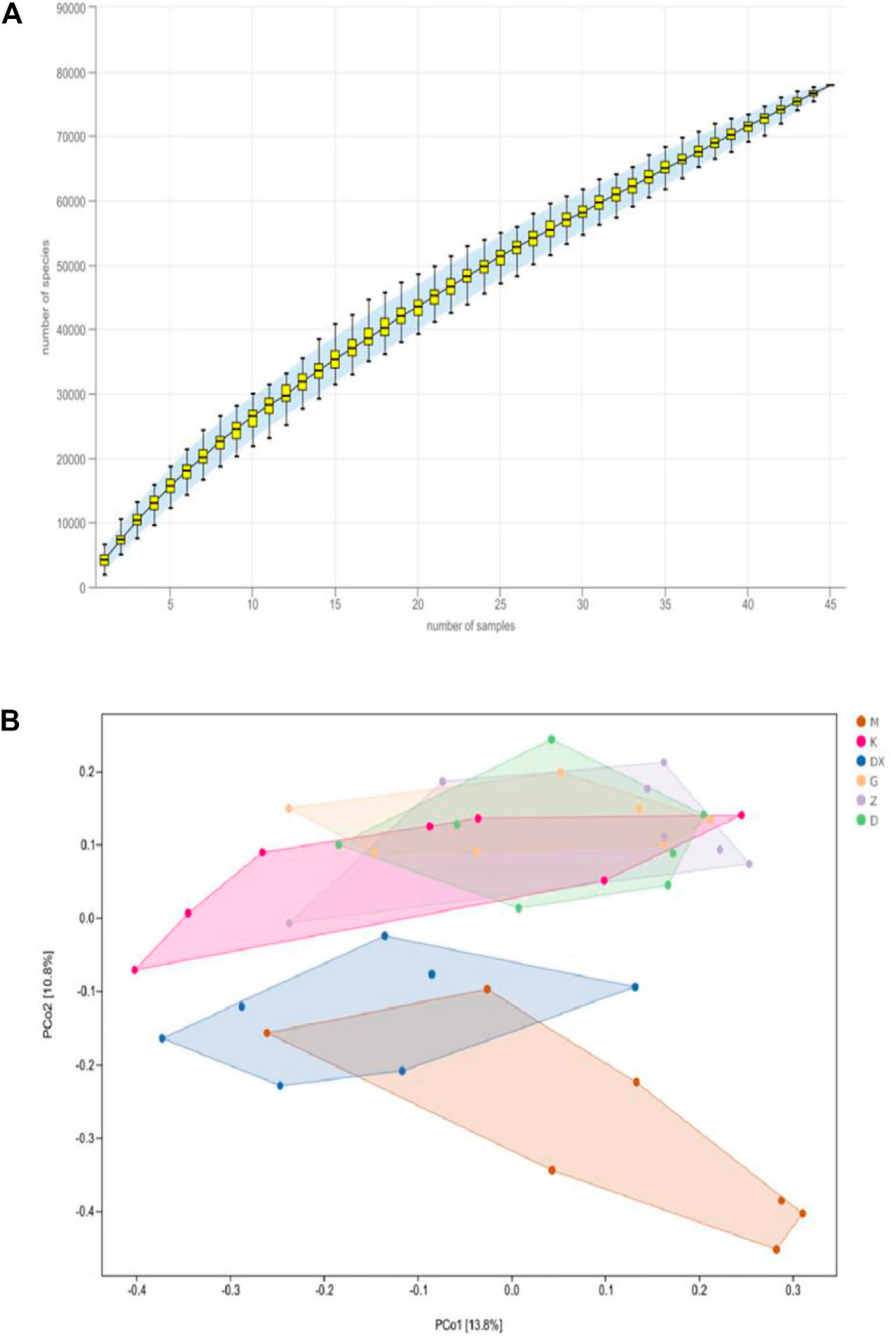
Effects of R-S on species accumulation curve **(A)** and principal coordinate analysis **(B)** of intestinal microflora in rats with insomnia (*n* = 7).

As shown in Figure 9A, the species accumulation curve showed that when the sample size was between 40 and 50, the species abundance in the community gradually stabilized, which confirms that the sample size was sufficient. As shown in Figure 9B, principal coordinate analysis (PCoA) showed that the composition of intestinal microflora in the model group significantly differed from the control group (*p* < 0.01). The composition of intestinal microflora in the R-S groups significantly differed from the model group (*p* < 0.01) but was similar to the control group. The intestinal microflora of the diazepam group deviated from the model group with a significant difference (*p* < 0.05).

### 3.6 Effects of R-S on the species classification composition of intestinal microflora in insomniac rats

**FIGURE10:**
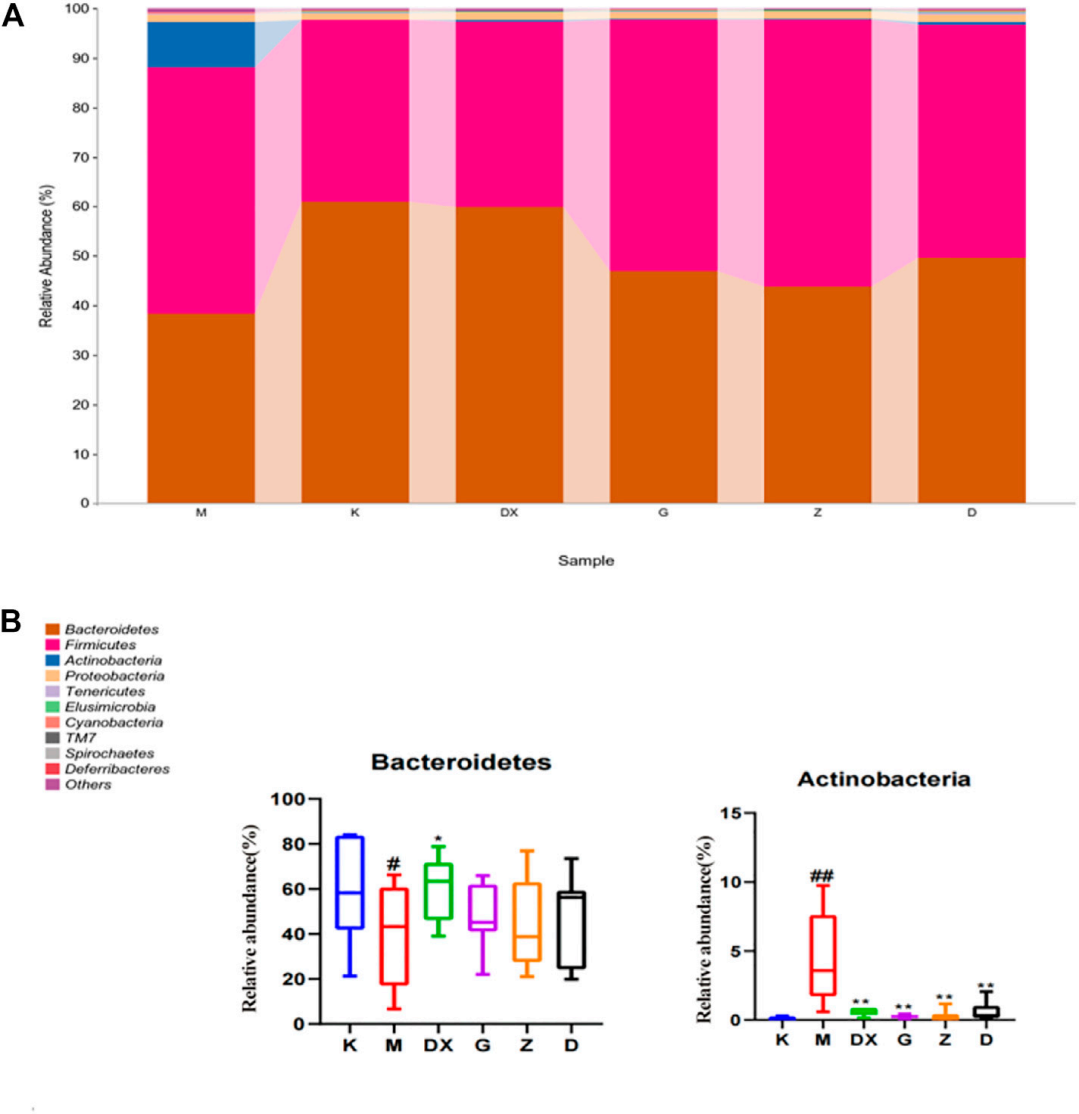
Effect of R-S on relative abundance of intestinal microflora **(A)** and significant changed intestinal microflora **(B)** of insomniac rats (*n* = 7) in phylum.

As shown in Table 2 and Figure 10, the intestinal microflora phyla mainly comprised Firmicutes, Bacteroidetes, Proteobacteria, and Actinobacteria. In this study, Firmicutes and Bacteroidetes were the most abundant in the intestinal tract of rats in each group. The proportions of Firmicutes, Bacteroidetes, Actinobacteria, and Proteobacteria in the intestinal microflora of rats in the control group were 36.65%, 60.95%, 1.25%, and 0.11%, respectively. The proportions of Firmicutes, Bacteroidetes, Proteobacteria, and Actinobacteria in the intestinal microflora of the model group were 49.68%, 38.42%, 1.49%, and 9.19%, respectively. Compared to the control group, the relative abundance of Bacteroidetes in the model group was lower (*p* < 0.05), and the relative abundance of Actinomycetes was significantly higher (*p* < 0.01). Compared with the model group, the relative abundance of Bacteroidetes in the intestinal tract of the diazepam group was higher (*p* < 0.05), the relative abundance of Bacteroidetes in the R-S groups was higher, while the relative abundance of Actinobacteria in the diazepam group was lower (*p* < 0.01). Meanwhile, the relative abundance of Actinobacteria in the R-S groups was significantly lower (*p* < 0.01). The F/B value of intestinal microflora (Firmicutes/Bacteroidetes) in the model group was higher than that in the control group, and the F/B value significantly decreased after RS intervention.

**TABLE 2:** Effects of R-S on species composition of the intestinal microflora of insomniac rats in phylum.

Phylum	K	M	DX	G	Z	D
Firmicutes	36.65%	49.68%	37.35%	50.80%	54.00%	46.97%
Bacteroidetes	60.95%	38.42%^#^	59.85%^*^	46.89%	43.71%	49.62%
Proteobacteria	1.25%	1.49%	1.40%	1.26%	1.34%	1.55%
Actinobacteria	0.11%	9.19%^##^	0.55%^**^	0.26%^**^	0.30%^**^	0.61%^**^
F/B	0.60	1.29	0.62	1.08	1.23	0.94

^#^ *p* < 0.05, ^##^ *p* < 0.01 vs. K; *p < 0.05, **p < 0.01 vs. M.

**FIGURE 11:**
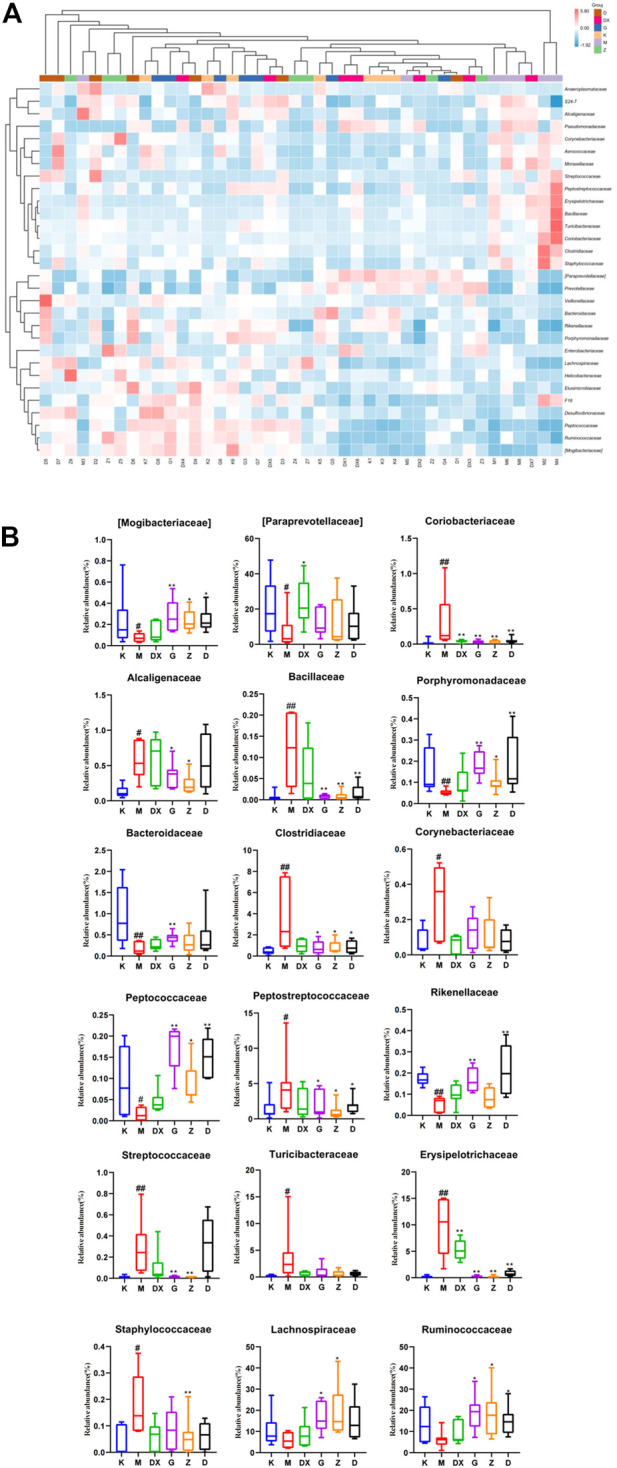
Effect of R-S on the relative abundance of intestinal microflora **(A)** and significant changed intestinal microflora **(B)** of insomniac rats (*n* = 7) in family.

Figure 11 displays, at the family level, the top 30 abundant intestinal microflora in the intestinal tract of rats in each group, indicating that the intestinal microflora structure of rats in the model group was significantly different from that in the control group. Compared with the control group, the relative abundance of Paraprevotellaceae, Bacteroidaceae, Mogibacteriaceae, Rikenellaceae, Porphyromonadaceae, and Peptococcaceae were lower (*p* < 0.05, *p* < 0.01), while the relative abundance of Ruminococcaceae and Lachnospiraceae was lower, but there was no significant difference. The relative abundance of Peptostreptococcaceae, Alcaligenaceae, Streptococcaceae, Bacillaceae, Erysipelotrichaceae, Clostridiaceae, Turicibacteraceae, Staphylococcaceae, Corynebacillaceae, and Coriobacteriaceae showed an upward trend (*p* < 0.05, *p* < 0.01). Compared with the model group, the relative abundance of Paraprevotellaceae was higher (*p* < 0.05), while the relative abundance of Coriobacteriaceae and Staphylococcaceae was lower in the intestinal tract of rats in the diazepam group (*p* < 0.05, *p* < 0.01). The relative abundance of Paraprevotellaceae in the intestinal tract of rats in the R-S groups was up-regulated without significant difference, and the relative abundance of Bacteroidaceae was up-regulated, especially in the intestinal tract of rats in the high-dose group (*p* < 0.01). The relative abundance of Ruminococcaceae and Lachnospiraceae was up-regulated (*p* < 0.05, *p* < 0.01) and there was a significant difference in the intestinal tract of rats in the high- and medium-dose groups (*p* < 0.05,*p* < 0.01). The relative abundance of Mogibacteriaceae, Porphyromonadaceae, and Peptococcaceae was higher (*p* < 0.05, *p* < 0.01). The relative abundance of Erysipelotrichaceae, Peptostreptococcaceae, Clostridiaceae, and Bacillaceae in the intestinal tract of rats in each dose group was lower (*p* < 0.05, *p* < 0.01). The relative abundance of Coriobacteriaceae, Alcaligenaceae, and Streptococcaceae in the intestinal tract of rats in the high- and medium-dose groups also showed a downward trend (*p* < 0.05, *p* < 0.01), while the relative abundance of Rikenellaceae in the intestinal tract of rats in the high- and low-dose groups decreased (*p* < 0.01). The relative abundance of Staphylococcaceae decreased, which was most obvious in the intestinal tract of rats in the medium-dose group (*p* < 0.01), while the relative abundance of Turicibacteraceae and Corynebacteriaceae showed a decreasing trend without significant difference.

**FIGURE 12:**
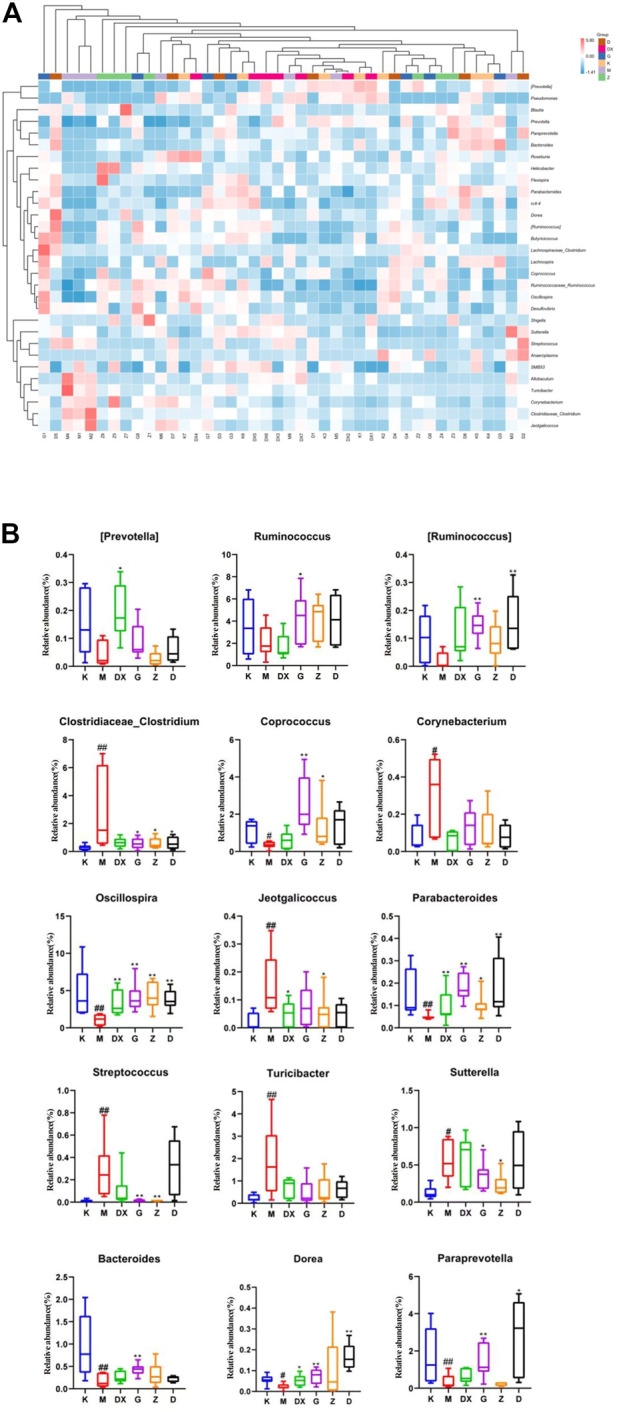
Effect of R-S on the relative abundance of intestinal microflora **(A)** and significant changed intestinal microflora **(B)** of insomniac rats (*n* = 7) in genus.

Figure 12 displays, at the genus level, the most abundant 30 intestinal microflora in the feces of rats in each group. Compared with the control group, the relative abundance of Paraprevotella, Bacteroides, Parabacteroides, Oscillospira, Coprococcus, Ruminococcus, and Dorea (*p* < 0.05, *p* < 0.01) in intestinal tract of rats in the model group was lower (*p* < 0.05, *p* < 0.01), and so was the relative abundance of Prevotella and Ruminococcus. However, there was no significant difference. The relative abundance of Clostridium, Turicibacter, Jeotgalicoccus, Corynebacterium, Allobaculum, Sutterella, and Streptococcus was higher (*p* < 0.05, *p* < 0.01). Compared with the model group, the relative abundance of Prevotella, Paraprevotella, Oscillospira, and Dorea in the intestinal tract of rats in the diazepam group was higher (*p* < 0.05, *p* < 0.01). The relative abundance of Oscillospira and Parabacteroides was higher in the intestinal tract of rats in the R-S groups (*p* < 0.01). The abundance of Prevotella was higher in the high- and medium-dose groups but there was no significant difference. The relative abundance of Ruminococcus, Paraprevotella, and Dorea in intestinal tract of rats in the R-S groups was significantly higher (*p* < 0.01), and the relative abundance of Coprococcus in the intestinal tract of rats in the high- and medium-dose group was higher (*p* < 0.05, *p* < 0.01). The relative abundance of Ruminococcus and Bacteroides in the intestinal tract of rats in the high-dose group was significantly increased (*p* < 0.05). The relative abundance of intestinal Allobaculum and Clostridium was significantly lower (*p* < 0.05, *p* < 0.01) in the intestinal tract of the rats in the R-S groups, and the relative abundance of intestinal Sutterella and Streptococcus was significantly lower (*p* < 0.05, *p* < 0.01) in the intestinal tract of the rats in the high- and medium-dose groups. The relative abundance of Jeotgalicoccus decreased most significantly in the medium-dose group (*p* < 0.05), while the relative abundance of Turicibacter and Corynebacterium in the R-S groups decreased without significant difference.

### 3.7 Effect of R-S on the metabolic pathway of insomniac rats

**FIGURE 13:**
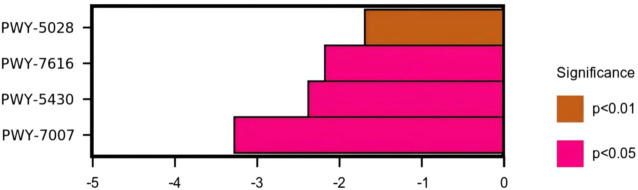
Differential analysis of the KEGG metabolic pathways.

The use of the KEGG database and metagenomeSeq method identified metabolic pathways with significant differences between groups (Table 3 and Figure 13). Compared with the model group, there were significant differences in the metabolic pathways of L-histidine degradation II (*p* < 0.01), methanol oxidation to carbon dioxide (*p* < 0.05), meta cleavage pathway of aromatic compounds (*p* < 0.05), and methyl ketone biosynthesis (*p* < 0.05) in the intestinal microflora of rats in the high-dose R-S group.

**TABLE 3:** Analysis of metabolic pathway differences of intestinal microflora.

ID of pathway	Name of pathway	logFC	SD	P
PWY-5028	L-histidine degradation II	−1.682	0.3994	0.009858
PWY-7616	methanol oxidation to carbon dioxide	−2.167	0.5769	0.02222
PWY-5430	meta cleavage pathway of aromatic compounds	−2.369	0.6573	0.03025
PWY-7007	methyl ketone biosynthesis	−3.268	0.8135	0.01137

## 4 Discussion

Insomnia is closely related to the disorder of neurotransmitter levels in the metabolic cycle of GLU/GABA-GLN (Liu et al., 2021). This cycle is the main pathway for the metabolic regulation of GLU and GABA, which is very important for maintaining GLU and GABA levels in the brain, which maintain its dynamic balance of excitation and inhibition system (Guo, 2019). GABA is an inhibitory neurotransmitter in the central nervous system, which is decarboxylated by GLU under the action of glutamate decarboxylase (GAD). The isoenzyme GAD65 of GAD is mainly responsible for synthesizing synaptically released GABA. GABA binds to receptors (GABAA, GABAB, GABAC) in the synaptic space (Lang and Chen, 2015). The GABAA receptor is commonly used in the study of insomnia, and the A receptors play further roles as the GABAARα1 receptors (Liu et al., 2011). Studies have shown that the content of GABA can be reduced in the hypothalamus and cerebral cortex, as can the expression of GABAAα1 positive cells in PCPA insomniac rats (Zhong et al., 2021). Shen et al. (2020) found that the content of GAD65 can be reduced in the brain of PCPA insomniac rats. GLU is an excitatory neurotransmitter in the central nervous system which is produced by glutamine (GLN) under the action of glutaminase. GLU is released in the synaptic space to bind to the receptor, having an excitatory role. About 20% of the neurons in the human brain are GLU neurons. GLU has an excitatory effect on neurons, but excessive GLU will produce neurotoxicity, leading to neuronal death and nervous system diseases (Machado-Vieira et al., 2012). Therefore, the remaining GLU in the synaptic space is transported by glutamate transporter to glial cells, where it is transformed into GLN by tricarboxylic acid cycle and under the action of glutamine synthetase (GS). It then enters the next cycle to avoid neurotoxicity caused by GLU accumulation.

The results showed that the level of GLU in the hypothalamus and brainstem of insomniac rats increased and the relative expression of GS protein decreased (Guo, 2019; Xing et al., 2021). GLU receptors are ion channel receptors (N-methyl aspartate receptor (NMDAR), α-amino-3-hydroxy-4-isooxy-propionic acid receptor (AMPAR), kainic acid receptor (KAR)), which are coupled with ion channels to form receptor channel complexes for rapid signal transduction. The other is a metabolic receptor (mGluRs) which is activated by coupling with G-protein in the membrane to produce slow physiological effects through the signal transduction system. mGluR5mRNA is widely distributed in the brain, playing a very important role in the regulation of the normal physiological functioning of the brain nervous system with its downstream signals. It participates in physiological processes such as the plastic changes of learning, memorizing, and synaptic transmission efficiency (Francesconi et al., 2004). Studies have found that mGluR5mRNA is associated with schizophrenia (Matosin et al., 2018), depression (Cosson et al., 2018), autism (D’Antoni et al., 2014), Parkinson’s (Vallano et al., 2013), Alzheimer’s (Zhang et al., 2019), and many other neurological diseases. There are three subtypes of NMDAR—NR1, NR2, and NR3—among which NR1 plays a more extensive role (Zhao et al., 2012). It was found that sleep demand was positively correlated with the transcriptional level of NMDAR1 in the brain area of the ventrolateral preoptic nucleus. The transcriptional level of NMDAR1 was lower when sleep pressure was low, and the transcription of NMDAR1 increased when sleep pressure was high (Wang, 2021). The AMPA receptor is a tetramer, composed of four sub-units (GluR1-4), which mediates rapid excitatory synaptic transmission in the central nervous system. McDermott et al. (2006) found that the expression of GluR1 (sub-unit of AMPAR) in the cortex and hippocampus increased after complete sleep deprivation.

The results showed that the GABA and GAD65 content and the expression of GABAAR α1 mRNA in the hippocampal tissue of the model group decreased. Compared with the model group, the GABA and GAD65 content in the hippocampus of the diazepam group and high- and medium-dose R-S groups increased while the expression of GABAARα1 mRNA decreased, which was close to that of the control group—especially the high-dose group. The GLU content in the hippocampus of rats in the model group increased, while the GLN and GS contents decreased. Compared with the model group, the GLU content in the hippocampus of rats in the diazepam group and the high- and medium-dose R-S groups decreased, while the GLN and GS contents increased, which was close to those of the control group—especially the high-dose group. The expressions of mGluR5mRNA, NR1mRNA, and GluR1mRNA in the hippocampus of rats in the model group all increased (*p* < 0.01). Compared with the model group, the expressions of mGluR5mRNA, NR1mRNA and GluR1mRNA in the hippocampus of rats in each group decreased, which was close to those in the control group.

The intestinal microflora plays an important role in human physiology and metabolic homeostasis. Under physiological conditions, the amount of Firmicutes and Bacteroidetes parasitic on the large intestine account for about 90% of the intestinal microflora, and the ratio between them is closely related to susceptibility to some diseases. Research demonstrates that the value of F/B is significantly increased after sleep deprivation (Benedict et al., 2016). Actinobacteria can produce bio-active substances in animal intestines, but they are conditional pathogens. For example, Corynebacterium, Mycobacteria, etc. can cause disease (Zhao et al., 2020). At the phylum level, this study found that Firmicutes increased, Bacteroidetes significantly decreased, and the F/B ratio increased significantly in insomniac rats. At the same time, it was found that the number of Actinobacteria in the intestinal tract of rats in the model group was significantly higher than in the control group. The diazepam and R-S groups could adjust the relative abundance of Firmicutes, Bacteroidetes, and Actinobacteria to make them close to those of the control group in order to maintain the homeostasis of intestinal microflora to regulate sleep.

At the family level, it was found that the relative abundance of Paraprevotellaceae, Bacteroidaceae, Mogibacteriaceae, Rikenellaceae, Porphyromonadaceae, and Peptococcaceae in the intestinal tract of insomniac rats decreased significantly, as did the relative abundance of Ruminococcaceae and Lachnospiraceae. However, there was no significant difference. On the other hand, the relative abundance of Peptostreptococcaceae, Alcaligenaceae, Streptococcaceae, Bacillaceae, Erysipelotrichaceae, Clostridiaceae, Turicibacteraceae, Staphylococcaceae, Corynebacteriaceae, and Coriobacteriaceae increased significantly. The relative abundance of beneficial bacteria such as Bacteroidaceae, Paraprevotellaceae, Ruminococcaceae, Lachnospiraceae, Rikenellaceae, and Porphyromonadaceae in the intestines of insomniac rats was all down-regulated. Tianwang Buxin Granule can play a role in calming the nerves by regulating the disordered intestinal flora of perimenopausal female insomniac patients, which is manifested by the decrease in the detection of *Barriella rosenbergii*, rumen coccus, Platycladus, and Clostridium saccharose in feces. The proportion of Clostridium praecox, Bacteroides, Bacteroides faecalis, Bifidobacterium and Lactobacillus increased (Yang et al., 2020). The acetic acid and lactic acid produced by beneficial bacteria can reduce intestinal Eh and PH values, improve the micro-environment of the intestine, protect the intestinal tract, and inhibit the growth of pathogenic bacteria. In addition, its metabolites have certain nutritional effects. The relative abundance of pathogenic or conditional pathogenic bacteria such as Peptostreptococcaceae, Alcaligenaceae, Streptococcaceae, Erysipelotrichaceae, Turicibacteraceae, Staphylococcaceae, Corynebacteriaceae, and Coriobacteriaceae increase in the intestinal tract of insomniac rats, which destroys the intestinal mucosal barrier, secretes a large amount of endotoxin, and increases intestinal permeability—further persecuting intestinal epithelial cells and causing intestinal inflammation. Zhao showed that Buzhong Yiqi Decoction can reduce the intestinal pathogenic bacteria of insomniac patients, increase the flora which maintains intestinal homeostasis, and improve the sleep state of insomniac patients with spleen deficiency (Zhao, 2020).

This study found that the relative abundance of beneficial bacteria such as Paraprevotellaceae, Bacteroidaceae, Rikenellaceae, and Porphyromonadaceae were up-regulated in each R-S group, especially in the high-dose group (*p* < 0.05, *p* < 0.01). However, the relative abundance of Peptostreptococcaceae, Alcaligenaceae, Streptococcaceae, Erysipelotrichaceae, Turicibacteraceae, Staphylococcaceae, Corynebacteriaceae, and Coriobacteriaceae decreased, which was close to those of the control group. The effect of high-dose R-S on pathogenic bacteria was particularly obvious (*p* < 0.05, *p* < 0.01). Although the relative abundance of beneficial bacteria in the intestinal tract of rats in the diazepam group showed an upward trend and the relative abundance of pathogenic bacteria decreased, there was no significant difference compared to the model group. All groups can thus promote the intestinal microflora of insomniac rats to return to a steady state, increasing the relative abundance of beneficial bacteria and reducing the relative abundance of pathogenic bacteria. However, the high-dose R-S group showed the best effect.

It was found that the relative abundance of Paraprevotella, Bacteroides, Parabacteroides, Oscillospira, Coprococcus, Ruminococcus, and Dorea decreased significantly in the intestinal tract of insomniac rats. The relative abundance of Prevotella and Ruminococcus decreased, but there was no significant difference. The relative abundance of Clostridium, Turicibacter, Jeotgalicoccus, Corynebacterium, Allobaculum, Sutterella, and Streptococcus increased significantly. The relative abundance of Prevotella in the intestines of patients with spleen deficiency and insomnia decreased significantly, while the relative abundance of Sutterella increased (Zhao, 2020). The relative abundance of Ruminococcus in the intestinal tract of insomniac patients with spleen-stomach disharmony decreased (Huang et al., 2019), which was consistent with the results of this experiment. A variety of neurotransmitters are produced in the intestinal tract, and intestinal microflora is the main producer of neurotransmitters (Huang et al., 2019). Strandwitz P et al. (2019) found that Bacteroides can produce a large amount of GABA. Liu Shujun et al. (2020) found that Bacteroides can secrete GAD and metabolize GLU to produce GABA. Olson et al. (2018) found that epilepsy can be treated by up-regulating the abundance of Parabacteroides and thus increasing the ratio of GABA/GLU. There is thus a positive correlation between Parabacteroides and the content of GABA.

In addition, it was found that the relative abundance of Bacteroides in the intestines of peri-menopausal insomniac patients decreased (Yang Y. W. et al., 2021). Thompson Robert S et al. (2021) found that an intake of probiotics for rats could increase the relative abundance of healthy microorganisms such as Parabacteroides, improve the intestinal micro-environment, and promote the recovery of sleep and a circadian rhythm. It can be inferred that Parabacteroides are closely related to sleep.

Chen Yiran et al. (2020) identified a positive correlation between Oscillospira and amount of sleep. Zhang et al. (2021) identified a negative correlation between Coprococcus, Dorea, and PSQI in the intestinal microflora of patients with severe depression associated with sleep disorder, demonstrating that Coprococcus and Dorea could promote sleep. Clostridium is a strict anaerobic bacillus containing Clostridium botulinum, C. tetanus and *C. perfringens*. Yu ShuYan et al. (2008) found that the application of C. tetanus neurotoxin could increase the expression of AMPAR on the cell surface. Animal experiments showed that the relative abundance of *C. perfringens* in the intestine of intermittent sleep-deprived mice increased significantly (Li et al., 2016), as did perfringens in the intestinal tract of sleep-deprived rats (Wang et al., 2017). Cui Xiaofang’s study found that the relative abundance of Clostridium and Turicibacter in insomniac patients increased significantly. Corynebacterium is a genus of Actinobacteria. Corynebacterium glutamate can be used to produce GLU (Long et al., 2019). As a central excitatory neurotransmitter, glutamate often leads to insomnia. In this study, it was found that the content of GABA in the hippocampi of insomniac rats decreased significantly, as did the relative abundance of Bacteroides and Parabacteroides in their intestinal tracts, further suggesting that the brain and intestine must be related and that intestinal microflora can affect sleep through neuroendocrine pathways. It was found that the relative abundance of Oscillospira in the model group decreased, which was consistent with the results of Chen Yiran et al. (2020).

The relative abundance of Coprococcus and Dorea decreased in the insomniac rats in the model group, which was consistent with Zhang Qi’s study. In this experiment, it was found that the relative abundance of Clostridium in the intestinal tract of the model group rats increased significantly, as did the expression of GluR1mRNA (sub-unit of AMPA receptor) in their hippocampi. Thus, the intestinal microflora can affect sleep by regulating the expression of neurotransmitter receptors. This experiment found that the relative abundance of Clostridium and Turicibacter increased significantly in insomniac rats, which was consistent with Cui Xiaofang’s study. In addition, the GLU content in the hippocampi of insomniac rats increased significantly, as did the relative abundance of Corynebacterium in the intestinal tract. The experiment found that, compared with the model group, all the treatment groups could adjust the abundance of bacteria to restore the intestinal microecological environment. It was found that the relative abundance of Prevotella, Paraprevotella, Oscillospira, and Dorea in the intestinal tract of rats in the diazepam group was significantly up-regulated, while Bacteroides, Parabacteroides, Ruminococcus, and Coprococcus were up-regulated with no significant difference. The relative abundance of Clostridium, Turicibacter, Jeotgalicoccus, Corynebacterium, Allobaculum, Sutterella, and Streptococcus decreased, but there was no statistical significance. The relative abundance of Oscillospira and Parabacteroides in the intestines of rats in the R-S groups increased significantly, and the relative abundance of Prevotella in the high- and low-dose groups increased, but not significantly. However, the relative abundance of Ruminococcus, Paraprevotella, and Dorea increased significantly, as did the relative abundance of Coprococcus, Ruminococcus and Bacteroides in the high-dose group. The relative abundance of Corynebacterium and Clostridium in each R-S group decreased significantly, the relative abundance of Sutterella and Streptococcus decreased significantly in the high- and medium-dose groups, and the relative abundance of Jeotgalicoccus decreased most significantly in the medium-dose group. The relative abundance of Turicibacteraceae and Corynebacterium decreased but not significantly. It can be seen from the above that the R-S groups had a better regulatory effect on the intestinal microflora of insomniac rats—especially the high-dose group. Consequently, applying R-S to improve the sleep condition of insomniac rats is closely related to the regulation of their intestinal microflora.

Through analysis of the KEGG functional metabolic pathway, it can be shown that high-dose R-S mainly affects the pathways of L-histidine degradation II, methanol oxidation to carbon dioxide, the meta cleavage pathway of aromatic compounds, and methyl ketone biosynthesis, in which the metabolic pathway of L-histidine degradation II may be related to the reduction of the relative abundance of histidine-producing bacteria in the intestinal tract of insomniac rats. As an important substance to promote awakening, histidine can excite the central nervous system to provoke insomnia. Yang Yuhan et al. (2020) found that one of the main ways that insomnia is caused by a heart–kidney imbalance is by unbalancing the body’s histidine metabolism . Studies have found that methanol can damage the central nervous system of humans, resulting in insomnia, dreamfulness, and blurred vision (Feng et al., 2004). Yan Jiao et al. (2019) found that methanol inhalation causes neurotoxicity and significant nerve cell apoptosis in rats. Therefore, the metabolic pathway of methanol oxidation to carbon dioxide may be related to high-dose R-S to reduce the relative abundance of methanol-producing bacteria. Both aromatic compounds and methyl ketones easily cause neurotoxicity and can harm the central nervous system of humans. This study found that the relative abundance of intestinal microflora involved in the meta cleavage pathway of aromatic compounds and methyl ketone biosynthesis metabolic pathways in insomniac rats in the high-dose R-S group was significantly lower than that in the model group.

Radix Ginseng and Semen Ziziphi Spinosae could affect the amount of GABA, GLN, GAD65, and GS and the expressions of mGluR5mRNA, NR1mRNA, and GluR1mRNA in the hippocampal tissue in rats induced by PCPA to insomnia through the GLU/GABA-GLN metabolic cycle. R-S can regulate the flora structure in the intestine of insomniac rats, increase beneficial intestinal bacteria, and reduce pathogenic bacteria to restore the homeostasis of the intestinal micro-environment. Therefore, the effect of R-S on the sleep of insomniac rats is closely related to the regulation of intestinal microflora. This experiment provides an experimental basis for potential action targets of insomnia treatment and the use of R-S in the treatment of insomnia.

## Data Availability

The original contributions presented in the study are included in the article/Supplementary Material; further inquiries can be directed to the corresponding author.
