# Supplementary material for: Effects of the Radix Ginseng and Semen Ziziphi Spinosae drug pair on the GLU/GABA-GLN metabolic cycle and the intestinal microflora of insomniac rats based on the brain–gut axis
Source: Front Pharmacol. 2022 Dec 21;13:1094507. doi: 10.3389/fphar.2022.1094507 (PMC9811267; doi:10.3389/fphar.2022.1094507)
Supplement: Supplementary file 1 [file DataSheet1.pdf]

DATA of Effects of Radix Ginseng and Semen Ziziphi Spinosae Drug Pair on the GLU/GABA-GLN Metabolic Cycle and Intestinal Microflora of Insomnia Rats Based on "Brain-Gut Axis"(1094507)

Contents

1 Family

2 Genus

3 Pathway

4 Phylum

5 GABA

6 GAD65

7 GLN

8 GLU

9 GS

10 GABAAR α 1

11 GluR-A

12 mGluR5

13 NR1

family

ID	M1	M2	M3	M4	M5	M6	M8	K1
S24-7	0.210736	0.139252	0.28082	0.047558	0.138281	0.352557	0.293147	0.126899
[Paraprev	0.010364	0.011606	0.111842	0.009263	0.29332	0.031915	0.103663	0.477724
Ruminococ	0.036902	0.065594	0.06085	0.01037	0.053855	0.141731	0.072264	0.044701
Lachnospi	0.096051	0.020952	0.048539	0.021691	0.060362	0.138335	0.10358	0.075144
Prevotell	0.030621	0.013867	0.066945	0.007399	0.175636	0.026706	0.162565	0.165625
Erysipelc	0.146353	0.150489	0.148654	0.357336	0.017	0.064693	0.054003	0.00072
Peptostre	0.041247	0.052271	0.033315	0.135916	0.040989	0.014044	0.010105	0.001356
Clostridi	0.074459	0.143947	0.027608	0.078707	0.018631	0.007425	0.008963	0.000614
Turicibac	0.025256	0.046425	0.023553	0.150442	0.008954	0.001496	0.006569	0.000254
Bacteroid	0.000523	0.001174	0.001263	0.000447	0.003615	0.000374	0.003647	0.012669
Desulfovi	0.00055	0.00113	0.002137	0.000835	0.001335	0.00259	0.004259	0.001949
Alcaligen	0.005208	0.005434	0.027147	0.001981	0.004153	0.008633	0.008824	0.00125
[Mogibact	0.00034	0.001391	0.001044	0.001204	0.000704	0.000317	0.000501	0.001102
Corynebac	0.004973	0.005216	0.00102	0.003593	0.000723	0.004892	0.000668	0.000297
Helicobac	0.000105	8.69E-05	0.001214	0.000175	0.001631	0.001324	0.002032	0.001017
Veillonel	0.000157	0.000174	0.000947	7.77E-05	0.000278	0.000518	0.000473	0.000275
Streptocc	0.002434	0.003977	0.001263	0.007943	0.000501	0.000691	0.004203	0.000169
Elusimicr	0.000314	0.000913	0.00102	0	0.000779	0.002245	0.000139	0.000508
Rikenella	0.000105	0.000239	0.000898	9.71E-05	0.000834	0.000806	0.000696	0.001674
Porphyrom	0.000628	0.000413	0.000826	0.000408	0.000519	0.000489	0.000501	0.000826
Coriobact	0.004004	0.010824	0.000753	0.017497	0.000501	0.001669	0.000612	0
Staphyloc	0.001937	0.008585	0.000826	0.002583	0.000797	0.003741	0.000807	0
Peptococc	0	2.17E-05	0.000121	0	0.000371	0.000317	0.000334	0.000254
Anaeropla	0	0	0.004638	0	0	0.000201	0.000362	0.000127
Enterobac	0.000105	6.52E-05	0.000486	5.83E-05	0.000185	0.000777	0	0
Aerococca	0.000654	0.00176	0.000777	0.000738	0.000704	0.001698	0.000724	0
F16	0.000157	0.002304	0.000146	0.001476	0.000148	0.000345	0.000139	0
Bacillace	0.002068	0.002065	0.001845	0.006408	0.000148	0.000345	0.000612	0
Pseudomon	0.000236	0	0	0.000952	0.00063	0.001698	0.001253	0.001123
Moraxella	0.000209	0.00113	0.000243	0.000233	0.00026	0.001727	0.000473	0.000127
Others	0.098171	0.072354	0.094724	0.0535	0.169055	0.161846	0.117582	0.083003

K2	K3	K4	K5	K6	K7	DX1	DX2	DX3
0.216107	0.195174	0.18416	0.226461	0.318892	0.101771	0.111512	0.149883	0.134182
0.145676	0.304633	0.33487	0.173888	0.017047	0.071072	0.446157	0.279916	0.164729
0.174794	0.048383	0.060289	0.123558	0.264045	0.21837	0.042732	0.06164	0.084863
0.080759	0.058101	0.037146	0.101632	0.098334	0.270588	0.030898	0.070917	0.084779
0.16208	0.249865	0.231039	0.115112	0.065686	0.029604	0.179891	0.242969	0.308735
0.00171	0.001026	0.000234	0.000797	0.00572	0.001465	0.043421	0.038107	0.08054
0.005873	0.006769	0.007353	0.021104	0.051288	0.020036	0.005316	0.011778	0.002675
0.00378	0.00359	0.001998	0.008621	0.008385	0.007079	0.002135	0.009345	0.003633
0.001553	0.001256	0.001424	0.003737	0.004957	0.00406	0.001935	0.008936	0.001245
0.003578	0.007743	0.016342	0.020406	0.004388	0.001801	0.00218	0.001592	0.004474
0.006188	0.001949	0.003591	0.005432	0.004859	0.019136	0.002603	0.001478	0.002775
0.000765	0.001538	0.000744	0.002915	0.012708	0.000459	0.007052	0.002024	0.00513
0.0027	0.000692	0.000383	0.001495	0.007622	0.003425	0.000378	0.000546	0.002506
0.000338	0.000333	0.000255	0.000922	0.001446	0.00196	0	0.000591	0
0.001125	0.000949	0.000191	0.000324	0.002795	0.0009	0.000578	0.000637	0.000202
0.001035	0.00041	0.000276	0.000698	0.002031	0.002524	0.001824	0.000432	0.004239
4.50E-05	0.000359	0.000128	0.000174	0.000114	7.06E-05	0.001513	0.000205	0.004407
0.00189	0.000462	0.000723	0.000972	0.003786	0.002136	0.000133	0.001432	0.000269
0.001485	0.001744	0.002274	0.001993	0.001528	0.001306	0.000845	0.000136	0.000959
0.0009	0.000769	0.001636	0.002666	0.003266	0.000583	0.000623	0.000114	0.001413
4.50E-05	0.000128	8.50E-05	9.97E-05	0.001089	0.000212	0	0.000227	0.000521
0	0	0	0.001071	0.000244	0.001147	0	0.00025	0
0.001058	0.000103	0.000128	0.000772	0.001771	0.002012	0.0004	0.000387	0.000252
0.0063	0	0	0.003214	0.000634	0.000141	0.000133	0	0.000101
0	0.000128	0.000234	0.000199	0.00065	0	0.004093	0	0.002103
4.50E-05	0.000128	0	0.000548	0.000439	0.002383	0	4.55E-05	0
0.000743	0.000282	0	0.000174	0.000845	0.001095	0	0.000159	0.000353
2.25E-05	0	0.000298	7.48E-05	0	7.06E-05	8.90E-05	0.000387	0
0.001035	0.000718	0.000213	0.001071	0.000861	0.000847	0.001602	0.001205	0.000202
6.75E-05	0	0.000276	0.000174	4.88E-05	0.001006	8.90E-05	0.000136	0
0.177471	0.112023	0.112544	0.178522	0.112131	0.23221	0.108953	0.105341	0.099077

DX4	DX5	DX6	DX7	G1	G3	G4	G5	G6
0.153447	0.333062	0.147594	0.217701	0.14019	0.1563	0.133977	0.102652	0.230337
0.14563	0.069872	0.350901	0.205436	0.031627	0.081436	0.216225	0.224232	0.137552
0.160382	0.17059	0.054208	0.057058	0.336623	0.122849	0.132455	0.072443	0.228721
0.212803	0.098012	0.052415	0.032998	0.259313	0.241731	0.128109	0.170181	0.124735
0.074641	0.038314	0.099365	0.104034	0.035584	0.157849	0.243068	0.271705	0.100509
0.029155	0.067512	0.057631	0.171112	0.005431	0.003879	0.002155	0.001185	0.001428
0.013808	0.044113	0.052641	0.041426	0.029816	0.046884	0.009744	0.0013	0.006334
0.008863	0.016807	0.016578	0.013693	0.014074	0.018427	0.004474	0.000554	0.006315
0.001867	0.010784	0.011341	0.010061	0.006833	0.034277	0.00317	0.000249	0.001278
0.002113	0.00409	0.001155	0.002775	0.004508	0.00647	0.004582	0.028105	0.00374
0.010094	0.003418	0.003588	0.003	0.009087	0.004082	0.002119	0.003346	0.002312
0.001703	0.009726	0.007341	0.008775	0.004437	0.007035	0.001684	0.001969	0.001767
0.002462	0.00177	0.000804	0.00049	0.005378	0.002938	0.001395	0.001319	0.002443
0.001087	0.001038	0.000845	0.001122	0.002112	0.000333	0.000453	0.000134	0.00141
0.000964	0.000224	0.001052	6.12E-05	0.001952	0.001896	0.001069	0.001587	0.000395
0.001662	0.001668	0.00099	0.000592	0.002076	0.001636	0.000543	0.001568	0.000752
0.000369	0.000183	0.000804	6.12E-05	8.87E-05	0	0.000272	0	0
0.004698	0.002747	0.000907	0.000388	0.000781	0.000159	0.001503	0.000134	0.002932
0.000759	0.001465	0.001629	0.001428	0.002272	0.002475	0.001069	0.001338	0.001673
0.000595	0.002381	0.000557	0.00151	0.001668	0.002736	0.001395	0.001702	0.001579
0.000472	0.000651	0.000454	0.00049	0.000532	0.000217	0.000254	0	0
0.000985	0.000692	0.00068	0.001469	0.000834	8.69E-05	0.000543	3.82E-05	0.001541
0.001067	0.002279	0.000268	0.000367	0.002165	0.001419	0.000761	0.001281	0.002124
0	0	0.000103	4.08E-05	0	0.000217	0.001485	0.000516	0.004417
0	0.000142	0.002619	2.04E-05	0.00055	7.24E-05	0.000833	0	0
0.00039	0.000488	0.000351	0.000959	0.000213	0.000101	0.000235	0	0.002105
0.000616	0.000244	0.000351	0.000327	0.001597	0.000492	0.000254	0.000249	0.000545
2.05E-05	0.000651	0.001237	0.001816	3.55E-05	0.000145	0.000127	0	1.88E-05
0.001046	0.00057	0.001196	0.001286	0	2.90E-05	0	0	0
0.000287	0.000326	0.000103	0.001735	0.000177	0	0	0	0.000263
0.164054	0.112764	0.121015	0.111707	0.099672	0.102584	0.104073	0.111619	0.130993

G7	G8	Z1	Z2	Z3	Z4	Z5	Z6	Z7
0.266073	0.194265	0.185588	0.141202	0.240923	0.228816	0.28621	0.107454	0.13662
0.065014	0.09215	0.007017	0.375198	0.137916	0.043453	0.007435	0.02211	0.024036
0.204392	0.193368	0.401227	0.160775	0.064887	0.219606	0.239446	0.176961	0.086139
0.163051	0.070885	0.131599	0.105106	0.096998	0.223179	0.1614	0.360958	0.431224
0.06022	0.148148	0.012567	0.086524	0.324434	0.140271	0.048378	0.1347	0.216236
0.001858	0.002521	0.002053	0.000686	0.000505	0.000596	0.001101	0.001529	0.005908
0.043141	0.006682	0.013289	0.009567	0.006147	0.00251	0.033973	0.004214	0.002778
0.012994	0.002335	0.013108	0.005031	0.004129	0.004062	0.019958	0.007586	0.004783
0.015796	0.001387	0.010874	0.002344	0.002477	0.000404	0.01761	0.004665	0.001565
0.004292	0.00225	0.000361	0.001239	0.007775	0.002403	0.005063	0.004293	0.002655
0.002448	0.019878	0.004963	0.00425	0.000895	0.00419	0.001492	0.015661	0.00109
0.004307	0.003806	0.005212	0.001487	0.002156	0.001234	0.003155	0.001274	0.001917
0.002507	0.004111	0.003181	0.001696	0.001193	0.002042	0.004109	0.003254	0.001565
0.002729	0.001929	0.003249	0.00162	0.000413	0.000425	0.010908	0.000451	0.000246
0.000118	0.002335	0.001015	0.000515	0.001881	0.002914	0.006628	0.012506	0.000475
0.001593	0.004432	0.000902	0.001372	0.000115	0.001212	0.000342	0.003646	0.000703
0.000265	8.46E-05	0	0.000191	0.000183	6.38E-05	0.000147	0.000137	5.28E-05
0.000973	0.000508	0.001827	0.002497	0.000528	0.000255	0.002519	0.000804	0.00116
0.001549	0.001133	0.000361	0.001334	0.001239	0.00034	0.001492	0.000745	0.000369
0.002478	0.000964	0.000429	0.00082	0.002087	0.001106	0.001076	0.000804	0.000809
2.95E-05	0.000711	0.000654	3.81E-05	4.59E-05	0.000128	0.000563	0.000137	0.000369
0.002094	0.001049	0.000745	0.000781	0.000482	0	0.002103	0.000255	5.28E-05
0.002021	0.001996	0.000902	0.000591	0.000596	0.001829	0.00044	0.001196	0.000615
0.000413	0.000677	0	0	6.88E-05	0	7.34E-05	0.000196	0
0.000826	0.001962	0.006114	0.000114	0.002729	0.000149	0.003057	0.000216	3.52E-05
0.001327	0.001303	9.02E-05	0.000457	9.18E-05	0	0.000856	0.000216	0
0.001224	0.001489	0.000835	0.000114	0.000138	0.000468	0.000587	0.000686	0.001037
0.000118	8.46E-05	4.51E-05	0.000114	4.59E-05	0	0.000318	0.000137	0
0.000147	0	0	0	0	0	0	0	0
0.000914	0.000829	0.000384	0.000191	0	0	0.000269	0.000118	0
0.133435	0.23348	0.190484	0.093823	0.098855	0.116896	0.138923	0.133034	0.07675

D1	D2	D3	D4	D5	D6	D7
0.145695	0.298746	0.357809	0.122409	0.146534	0.245866	0.170578
0.330413	0.151793	0.051071	0.027647	0.179713	0.102236	0.024226
0.074907	0.150372	0.140283	0.278416	0.092506	0.145816	0.183391
0.073246	0.066008	0.07919	0.177743	0.185075	0.135839	0.323431
0.225471	0.105688	0.117357	0.041649	0.2005	0.194272	0.039744
0.003459	0.0167	0.013943	0.002677	0.008475	0.003055	0.006715
0.007418	0.020353	0.042918	0.019714	0.015473	0.009388	0.019552
0.004027	0.007468	0.016877	0.015014	0.002154	0.002803	0.012813
0.001616	0.012003	0.009918	0.007021	0.006714	0.002298	0.005007
0.002526	0.002915	0.002726	0.001349	0.023728	0.015554	0.001637
0.002662	0.005183	0.001895	0.010868	0.010189	0.004372	0.009847
0.002139	0.010815	0.009555	0.000992	0.007092	0.004932	0.001874
0.001274	0.001692	0.002622	0.004562	0.003082	0.00213	0.002112
0.001365	0.001692	0.000675	0.000853	0.00022	0.00014	0.005576
0.000273	0.002447	0.001714	0.001507	0.003223	0.001289	0.000807
0.000341	0.000756	0.000883	0.001805	0.015158	0.000645	0.002325
0.002913	0.012111	0.003791	0.000139	0.006746	0.000757	0.005149
0.001115	0.000936	0.00013	0.007497	0.000692	0.007987	0.00083
0.001001	0.002519	0.001973	0.001388	0.003318	0.003811	0.000854
0.00091	0.002159	0.001168	0.001011	0.003161	0.00412	0.000546
0.000137	0.00135	0.000545	0.000456	0.000299	0.000196	0.000522
0.001047	0.000828	0.000493	0.001289	0	0.000112	0.003369
0.001001	0.001512	0.001766	0.001944	0.001195	0.002186	0.000997
0.001934	0.007558	7.79E-05	0.000992	0	0	0.002041
0.000137	7.20E-05	0.000208	0	0.000189	0	0
0.000796	0.001242	2.60E-05	0.000357	3.15E-05	0.000112	0.003939
0.00025	0.000828	0.001013	0.001765	0.000142	0.000252	0.000522
6.83E-05	0.000234	0.000312	0.000535	0	2.80E-05	7.12E-05
0	0	0.000156	0	0	0.000813	0.000427
0.000432	0.001242	0.000104	0.000357	0	8.41E-05	0.002041
0.109425	0.095161	0.135817	0.267805	0.081876	0.10885	0.164598

genus

ID	M1	M2	M3	M4	M5	M6	M8	K1
[Prevotel	0.009553	0.010867	0.109754	0.008117	0.284496	0.029843	0.092083	0.470076
Prevotell	0.024497	0.01278	0.045456	0.006719	0.132089	0.01131	0.140018	0.103976
Oscillosp	0.001492	0.00915	0.018284	0.00266	0.015851	0.011856	0.018985	0.019681
Blautia	0.082598	0.008216	0.004007	0.013535	0.001817	0.004058	0.063022	0.026884
Allobacul	0.145437	0.150098	0.148241	0.356539	0.016926	0.064462	0.053864	0.000127
Ruminococ	0.011987	0.034536	0.015176	0.003029	0.017538	0.04544	0.02458	0.005847
Paraprevc	0.000811	0.000739	0.001578	0.001126	0.006711	0.001871	0.010689	0.002796
Coprococc	0.000183	0.002326	0.003497	0.003573	0.005339	0.004374	0.005651	0.004004
Clostridi	0.059384	0.131167	0.019207	0.070007	0.011272	0.005612	0.00451	0.000381
Turicibac	0.025256	0.046425	0.023553	0.150442	0.008954	0.001496	0.006569	0.000254
Roseburia	0.000942	0.001022	0.006556	0.000252	0.008695	0.019684	0.002839	0.001504
Bacteroid	0.000523	0.001174	0.001263	0.000447	0.003615	0.000374	0.003647	0.012669
Sutterell	0.005182	0.005173	0.027123	0.001981	0.003875	0.008403	0.008824	0.00125
SMB53	0.006072	0.001608	0.002598	0.001573	0.003652	0.000547	0.002477	0.000233
Corynebac	0.004973	0.005216	0.00102	0.003593	0.000723	0.004892	0.000668	0.000297
Streptocc	0.002434	0.003564	0.001263	0.007787	0.000501	0.000691	0.004203	0.000169
Lachnospi	0.000393	0	7.29E-05	0.000272	0.000426	0.000115	0.003034	0.001356
Parabacte	0.000471	0.000391	0.000801	0.000408	0.000501	0.00046	0.000501	0.000826
[Ruminocc	0	0	0.000704	1.94E-05	0.00013	0	0.000501	0.000678
Dorea	0	0.000196	0.000194	0.00035	0.000148	0.000489	0.000306	0.000551
Helicobac	0	0	0.000777	0	0.00089	0.001324	0.001503	0.000826
Anaeropla	0	0	0.004638	0	0	0.000201	0.000362	0.000127
Jeotgalic	0.001387	0.006868	0.000704	0.002117	0.000779	0.003482	0.000585	0
Shigella	0.000105	6.52E-05	0.000219	5.83E-05	5.56E-05	0.000719	0	0
rc4-4	0	0	0	0	0.000241	0	0	0.000148
Flexispir	0.000105	8.69E-05	0.000437	0.000175	0.000742	0	0.000529	0.000191
Desulfovi	0.000471	0.000543	0.000243	0.000699	7.42E-05	0.000173	0.000223	2.12E-05
Pseudomon	0.000236	0	0	0.000952	0.00063	0.001669	0.001197	0.001123
Butyricic	0	8.69E-05	0	9.71E-05	0	0.000547	0	0.000699
Lachnospi	0	0	0	0	0.000167	0.001151	0.000724	0
Others	0.410689	0.331776	0.508195	0.282358	0.468067	0.750899	0.511608	0.342711

K2	K3	K4	K5	K6	K7	DX1	DX2	DX3
0.131343	0.279377	0.296066	0.129164	0.013326	0.060903	0.438906	0.275187	0.153224
0.141423	0.213841	0.168052	0.098542	0.061721	0.026144	0.133978	0.205907	0.294538
0.073086	0.019846	0.022505	0.036153	0.057788	0.108726	0.021377	0.019304	0.051876
0.010148	0.007308	0.002826	0.009742	0.026196	0.000159	0.005606	0.004502	0.026611
6.75E-05	0.000538	0	0.000623	0.004128	0.000177	0.043243	0.038062	0.079026
0.048536	0.009974	0.010668	0.033537	0.06814	0.060356	0.006963	0.01296	0.011539
0.012488	0.021076	0.032216	0.040115	0.003591	0.009762	0.00525	0.003843	0.010799
0.016449	0.017282	0.002401	0.01642	0.005672	0.013752	0.005962	0.001228	0.014012
0.003645	0.001359	0.000871	0.003015	0.004306	0.006443	0.001846	0.005139	0.00323
0.001553	0.001256	0.001424	0.003737	0.004957	0.00406	0.001935	0.008936	0.001245
0.000428	0.002282	0.006141	0.014302	0.006143	0.043515	0.001535	0.010118	0.012414
0.003578	0.007743	0.016342	0.020406	0.004388	0.001801	0.00218	0.001592	0.004474
0.000765	0.001538	0.000744	0.002915	0.012497	0.000459	0.007052	0.001955	0.00513
0	0.002231	0.001126	0.005407	0.003575	0	0	0.001955	0.000404
0.000338	0.000333	0.000255	0.000922	0.001446	0.00196	0	0.000591	0
4.50E-05	0.000333	0.000128	0.000174	0.000114	7.06E-05	0.001513	0.000205	0.004407
0.000203	0.000462	0.000319	0.000274	0.000114	0.000741	0.000156	0.00025	5.05E-05
0.0009	0.000769	0.001636	0.002666	0.003234	0.000583	0.000623	0.000114	0.001413
0.001035	2.56E-05	0.000106	0.001196	0.002178	0.001818	0.000601	0.000205	0.000942
0.000923	0.000462	0.000128	0.000523	0.000683	0.000724	0.0002	0.000637	0.000286
0.000383	0.000513	0.000191	0.000324	0.000423	0.000759	0.000156	0.000432	8.41E-05
0.0063	0	0	0.003214	0.000634	0.000141	0.000133	0	0.000101
0	0	0	0.000548	0.000244	0.000706	0	0.000205	0
0	0	0.000234	4.98E-05	0.000634	0	0.002536	0	0.001951
0.000405	0	0.000128	0.000349	0.001365	0.000741	0.000178	0.000227	0.000135
0.000743	0.000436	0	0	0.002373	0.000141	0.000423	0.000205	0.000118
0.00099	7.69E-05	6.38E-05	0	0.000699	0.000318	4.45E-05	0.000273	0.000303
0.001035	0.000692	0.000128	0.001046	0.000861	0.000847	0.001602	0.001205	0.000202
0.000833	0	0	0.000174	0.001024	0.000477	0	0.000182	0
0	0	0.000914	0.000897	0.000325	0.000388	0	0.000136	0
0.541527	0.409502	0.433219	0.572393	0.704835	0.652797	0.313091	0.395262	0.315851

DX4	DX5	DX6	DX7	G1	G3	G4	G5	G6
0.144009	0.066311	0.338447	0.192988	0.029355	0.05609	0.204488	0.205839	0.125298
0.059807	0.027245	0.065797	0.079913	0.033224	0.153043	0.217511	0.254842	0.082185
0.0603	0.043441	0.026001	0.017428	0.139427	0.039618	0.029596	0.032675	0.021463
0.005252	0.028344	0.003505	0.004592	0.077558	0.144199	0.044973	0.0456	0.049465
0.028519	0.066637	0.057508	0.170888	0.001136	0.001476	0.001503	0.000459	0.000319
0.037998	0.026756	0.010186	0.01004	0.054965	0.016965	0.044067	0.018718	0.045105
0.001518	0.003337	0.011011	0.010999	0.002201	0.024709	0.010686	0.014072	0.011164
0.005437	0.006755	0.001175	0.009734	0.039862	0.009206	0.019924	0.019196	0.02135
0.006319	0.011984	0.009299	0.008551	0.009335	0.011623	0.0023	0.000554	0.005356
0.001867	0.010784	0.011341	0.010061	0.006833	0.034277	0.00317	0.000249	0.001278
0.039988	0.002442	0.002041	0	0.012459	0.007107	0.001123	0.003136	0.0053
0.002113	0.00409	0.001155	0.002775	0.004508	0.00647	0.004582	0.028105	0.00374
0.001703	0.009665	0.007341	0.008122	0.004437	0.007035	0.001503	0.001969	0.001767
0.001334	0.003785	0.005485	0.003979	0.003851	0.006036	0.002065	0	0.000958
0.001087	0.001038	0.000845	0.001122	0.002112	0.000333	0.000453	0.000134	0.00141
0.000349	0.000183	0.000804	6.12E-05	8.87E-05	0	0.000272	0	0
0.000164	0.000488	0.000124	0.000347	0.016754	0.000391	0.004673	0.000421	0.002594
0.000595	0.00234	0.000557	0.00151	0.001668	0.002736	0.001395	0.001702	0.001579
0.000698	0.002136	0.002845	0.000531	0.002272	0.001534	0.001684	0.001396	0.000639
0.002708	0.000671	0.000969	0.000429	0.001171	0.001057	0.000724	0.000803	0.000357
0.000964	0.000224	0.00068	6.12E-05	0.001278	0.000449	0.000362	0.001109	0.000263
0	0	0.000103	4.08E-05	0	0.000217	0.001485	0.000516	0.004417
0.000882	0.000651	0.000536	0.001163	0.000692	8.69E-05	0.00038	3.82E-05	0.001372
0	0.000142	0.001732	2.04E-05	0.000515	7.24E-05	0.000761	0	0
0.000431	0.001974	0.000144	0.000224	0.001597	0.001071	0.000598	0.001013	0.001635
0	0	0.000371	0	0.000674	0.001447	0.000543	0.000363	0.000132
0.001785	0.000163	0.000227	0.000449	0.002236	0.000579	0.000181	0.00044	0.000413
0.001046	0.000549	0.001196	0.001265	0	2.90E-05	0	0	0
0.000349	0.000529	0.000309	0	0.00197	0.000999	0.000543	0	0.000207
0.000267	0	0.001196	0	0.002059	0	0.000851	0.00174	0
0.588552	0.673917	0.427935	0.456646	0.54539	0.469899	0.395628	0.364319	0.60845

G7	G8	Z1	Z2	Z3	Z4	Z5	Z6	Z7
0.05584	0.06449	0.005009	0.373654	0.073236	0.040029	0.006188	0.020797	0.020467
0.053495	0.14204	0.010784	0.057517	0.302576	0.127446	0.045297	0.131329	0.207462
0.040929	0.079428	0.062269	0.039774	0.015253	0.033329	0.043609	0.066252	0.029961
0.027123	0.005041	0.014123	0.035715	0.041767	0.110813	0.053686	0.014936	0.376787
0.001077	0.000406	0.001286	7.62E-05	0.000138	0	0.000245	0.000176	0.00473
0.059055	0.078565	0.052455	0.021116	0.016789	0.054832	0.064203	0.048631	0.023807
0.00879	0.026848	0.001985	0.001029	0.062134	0.003297	0.00115	0.001137	0.00327
0.049468	0.014109	0.015161	0.0081	0.003876	0.018228	0.005307	0.038105	0.004554
0.008186	0.002318	0.009521	0.002535	0.0025	0.003169	0.012792	0.006958	0.004308
0.015796	0.001387	0.010874	0.002344	0.002477	0.000404	0.01761	0.004665	0.001565
0.004262	0.00159	0.005753	0.01092	0.007363	0.018419	0.004402	0.002607	0.000422
0.004292	0.00225	0.000361	0.001239	0.007775	0.002403	0.005063	0.004293	0.002655
0.004203	0.003739	0.005212	0.001487	0.002156	0.001234	0.003131	0.001156	0.001917
0.004543	0	0.002775	0.00242	0.001468	0.000787	0.006163	0.000627	0.000475
0.002729	0.001929	0.003249	0.00162	0.000413	0.000425	0.010908	0.000451	0.000246
0.000265	8.46E-05	0	0.000191	0.000183	6.38E-05	0.000147	0.000137	5.28E-05
0.004469	8.46E-05	0.000226	0.002973	0.001812	0.000957	0.000978	0.000294	0.000193
0.002478	0.000964	0.000429	0.00082	0.002087	0.001106	0.001076	0.000804	0.000809
0.001327	0.004991	0.001444	0.00082	2.29E-05	0.000447	0.00115	0.00198	0.000545
0.000929	0.00022	0.000654	0	0.000459	0.000659	0.000514	7.84E-05	0.003816
0.000118	0.002047	0.000812	0.000515	0.001239	0.001787	0.006628	0.007037	8.79E-05
0.000413	0.000677	0	0	6.88E-05	0	7.34E-05	0.000196	0
0.002006	0.000964	0.000745	0.000686	0.000482	0	0.00181	0.000176	0
0.000782	0.001861	0.005956	9.53E-05	0.002569	0	0.002788	0.000137	0
0.001622	0.000389	0.000384	0.000172	0.000505	0.00134	0.000318	0.000412	0.000422
0	0.000288	0.000203	0	0.000642	0.001127	0	0.005312	0.000299
0.000339	0.002165	0.000564	0.000248	0.000138	0.000425	0	0.00096	0.000299
2.95E-05	0	0	0	0	0	0	0	0
0.000383	0.0011	0.000564	0.000896	0.000115	0.000447	0.000391	0.000764	0.000141
0	0.000372	0	0.00101	0	0.000553	0.000342	0	0
0.643457	0.556403	0.786278	0.431781	0.449758	0.574826	0.703664	0.639532	0.309901

D1	D2	D3	D4	D5	D6	D7
0.32345	0.099696	0.014722	0.021023	0.13331	0.068858	0.02107
0.206653	0.090248	0.111878	0.040935	0.19844	0.189311	0.037774
0.019296	0.036999	0.033831	0.102913	0.032706	0.058377	0.046957
0.001115	0.01463	0.003064	0.003808	0.089188	0.067205	0.014972
0.003072	0.015674	0.013631	0.001825	0.004576	0.000589	0.00624
0.017817	0.041318	0.068181	0.063942	0.019718	0.016563	0.041761
0.005256	0.050658	0.035622	0.006426	0.046308	0.032201	0.003108
0.017452	0.003419	0.017032	0.026596	0.007752	0.002046	0.022257
0.003732	0.005255	0.012073	0.010789	0.00184	0.001177	0.006857
0.001616	0.012003	0.009918	0.007021	0.006714	0.002298	0.005007
0.008715	0.006029	0.001039	0.016184	0.00607	0.006334	0.035971
0.002526	0.002915	0.002726	0.001349	0.023728	0.015554	0.001637
0.002116	0.010815	0.009555	0.000992	0.007092	0.004932	0.00178
0	0.001728	0.002674	0.00357	0	0.001625	0.004081
0.001365	0.001692	0.000675	0.000853	0.00022	0.00014	0.005576
0.002913	0.011859	0.003791	0.000139	0.006746	0.000757	0.005149
0.000159	0.000144	0	0.000278	0.006777	0.000336	0.001163
0.00091	0.002159	0.001168	0.001011	0.003145	0.004064	0.000546
0.000614	0.001566	0.000623	0.003272	0.006761	0.001149	0.002278
0.001684	0.001404	0.001194	0.000972	0.007673	0.00269	0.002041
0.000273	0.00117	0.000675	0.001368	0.001054	0.001037	0.000569
0.001934	0.007558	7.79E-05	0.000992	0	0	0.002041
0.000751	0.000792	0.000338	0.001051	0	0	0.002824
0.000137	7.20E-05	0.000208	0	0.000189	0	0
0.000523	0.000864	0.00109	0.000873	0.000865	0.001962	0.000285
0	0.001278	0.001039	0.000139	0.00217	0.000252	0.000237
0.000205	0.000738	0.000779	0.000714	0.000676	0.000336	0.000878
0	0	0.000156	0	0	0.000813	0.000403
0.000228	0.000432	0.000467	0.000714	0.001887	0.00042	0.000403
0.000273	5.40E-05	0	0.00123	0.000472	0.000953	0
0.373214	0.559215	0.648786	0.678785	0.381408	0.517964	0.721675

pathway	descriptilogFC		SE	Pvalues	adjPvalues
3-HYDROXY	4-hydroxy	1.564	1.244	0.2088	0.8498
P341-PWY	glycolysi	1.262	1	0.2069	0.8498
PWY-7013	L-1,2-pro	1.167	0.5864	0.04657	0.6049
PWY-7237	myo-, chi	1.136	0.5053	0.0246	0.4534
P163-PWY	L-lysine	1.107	0.5341	0.0382	0.5834
PWY-6478	GDP-D-gly	1.106	1.158	0.3397	0.8498
GLUCARDEG	D-glucara	1.084	0.6745	0.1079	0.8498
PWY-5677	succinate	1.062	0.6232	0.08825	0.8498
NAD-BIOSY	NAD salva	1.059	0.5242	0.04342	0.6001
GALACTARD	D-galacta	1.055	0.6367	0.09759	0.8498
GLUCARGAL	superpath	1.055	0.6367	0.09759	0.8498
PWY-6749	CMP-legio	0.9977	0.502	0.04689	0.6049
FUC-RHAMC	superpath	0.9424	0.4374	0.03121	0.5251
PWY-7446	sulfoglyc	0.9332	0.958	0.33	0.8498
PWY-7374	1,4-dihyd	0.9321	0.6875	0.1752	0.8498
PWY-7371	1,4-dihyd	0.9259	0.7069	0.1902	0.8498
PWY-6629	superpath	0.9081	0.9739	0.3511	0.8498
PWY-5676	acetyl-Co	0.9012	0.4301	0.03613	0.5827
PWY-6263	superpath	0.8867	0.6748	0.1888	0.8498
KETOGLUCO	ketogluco	0.8803	0.8915	0.3234	0.8498
LPSSYN-PW	superpath	0.8697	0.9777	0.3737	0.8498
PWY-7332	superpath	0.8209	0.7657	0.2837	0.8498
PWY-5705	allantoin	0.8198	0.3976	0.03919	0.5834
ECASYN-PW	enterobac	0.7672	0.9402	0.4145	0.8498
PWY-6641	superpath	0.7511	0.771	0.33	0.8498
PWY-6572	chondroit	0.7174	0.8915	0.421	0.8498
REDCITCYC	TCA cycle	0.6917	0.3904	0.07646	0.8354
PWY-6588	pyruvate	0.6901	0.4133	0.09501	0.8498
FUCCAT-PW	fucose de	0.6844	0.6427	0.287	0.8498
P162-PWY	L-glutama	0.6763	0.6874	0.3252	0.8498
PWY-7315	dTDP-N-ac	0.6738	0.5369	0.2094	0.8498
P562-PWY	myo-inosi	0.6575	0.4871	0.1771	0.8498
PWY-7328	superpath	0.6557	0.6594	0.32	0.8498
PWY-1622	formaldeh	0.644	0.3327	0.05293	0.6401
PWY-5177	glutaryl-	0.6367	0.5488	0.2459	0.8498
PWY-5971	palmitate	0.6284	0.6773	0.3535	0.8498
PWY-6282	palmitole	0.6169	0.8476	0.4667	0.8498
PWY-5989	stearate	0.6158	0.8543	0.471	0.8498
FASYN-INI	superpath	0.6153	0.8572	0.4728	0.8498
PWY-7664	oleate bi	0.5986	0.8214	0.4662	0.8498
GALACTURC	D-galactu	0.5896	0.4045	0.145	0.8498
GALACT-GL	superpath	0.5874	0.4166	0.1586	0.8498
GLUCUROCA	superpath	0.5758	0.409	0.1591	0.8498
PWYG-321	mycolate	0.5729	0.8148	0.482	0.8498
PWY-6467	Kdo trans	0.5441	0.6768	0.4215	0.8498
NAGLIPASY	lipid IVA	0.5427	0.679	0.4241	0.8498
PWY0-862	(5Z)-dode	0.5406	0.7983	0.4983	0.8498
ORNDEG-PW	superpath	0.5241	0.3132	0.09424	0.8498
PWY-7373	superpath	0.5139	1.051	0.6249	0.8762
PWY-5531	chlorophy	0.5134	0.1928	0.007754	0.2

PWY-7159	chlorophy	0.5134	0.1928	0.007754	0.2
PWY-7198	pyrimidin	0.5103	0.896	0.569	0.8498
PWY-6471	peptidogl	0.5055	0.3634	0.1641	0.8498
PWY0-1241	ADP-L-gly	0.5053	0.5684	0.374	0.8498
PWY-7210	pyrimidin	0.5022	0.8401	0.55	0.8498
PWY-1269	CMP-3-dec	0.5016	0.6455	0.4371	0.8498
PWY-5304	superpath	0.4927	0.59	0.4037	0.8498
PWY-6519	8-amino-7	0.4892	0.6824	0.4734	0.8498
FAO-PWY	fatty aci	0.4503	0.4409	0.3071	0.8498
COBALSYN-	adenosylc	0.4393	0.2952	0.1367	0.8498
BIOTIN-BI	biotin bi	0.4343	0.6024	0.471	0.8498
PWY-7323	superpath	0.432	0.4894	0.3774	0.8498
PWY-6269	adenosylc	0.428	0.2974	0.15	0.8498
PWY-5509	adenosylc	0.4248	0.2983	0.1544	0.8498
PWY-7254	TCA cycle	0.4205	0.5179	0.4168	0.8498
PWY0-1586	peptidogl	0.4153	0.3299	0.2081	0.8498
PWY-6703	preQ0 bic	0.4024	0.4421	0.3627	0.8498
PWY-6507	4-deoxy-L	0.4001	0.4816	0.4061	0.8498
PWY-5741	ethylmalc	0.3811	0.6319	0.5464	0.8498
KDO-NAGLI	superpath	0.3728	1.214	0.7587	0.9411
PWY0-1533	methylphc	0.3716	0.8965	0.6785	0.915
CENTFERM-	pyruvate	0.3648	0.4468	0.4142	0.8498
PWY-6590	superpath	0.3589	0.4402	0.4149	0.8498
FASYN-ELC	fatty aci	0.3534	0.3451	0.3059	0.8498
METHGLYUT	superpath	0.349	0.9055	0.6999	0.9163
PWY-7242	D-fructur	0.3394	0.3352	0.3113	0.8498
SO4ASSIM-	sulfate r	0.3317	0.3709	0.3712	0.8498
SULFATE-C	superpath	0.3306	0.3524	0.3482	0.8498
HISDEG-PW	L-histidi	0.322	0.5564	0.5627	0.8498
PWY-7090	UDP-2,3-d	0.3211	0.556	0.5636	0.8498
P42-PWY	incomplet	0.308	0.3905	0.4303	0.8498
PYRIDNUCS	NAD biosy	0.2916	0.3008	0.3324	0.8498
GLYCOL-GL	superpath	0.2908	0.3396	0.3918	0.8498
SER-GLYSY	superpath	0.2902	0.2821	0.3035	0.8498
PWY-6969	TCA cycle	0.2889	0.3241	0.3727	0.8498
PWY-7663	gondoate	0.287	0.2636	0.2762	0.8498
PWY-5973	cis-vacce	0.2853	0.2674	0.2859	0.8498
GLUCONEO-	gluconeog	0.2827	0.2961	0.3396	0.8498
THISYN-PW	superpath	0.2779	0.3453	0.4209	0.8498
TRPSYN-PW	L-tryptop	0.2773	0.2787	0.3197	0.8498
CALVIN-PW	Calvin-Be	0.2728	0.2816	0.3326	0.8498
PWY-6700	queuosine	0.2718	0.3612	0.4517	0.8498
COLANSYN-	colanic a	0.2693	0.4453	0.5453	0.8498
PWY-5154	L-argininr	0.2601	0.4236	0.5391	0.8498
POLYISOPR	polyisopr	0.247	0.3588	0.4913	0.8498
ALL-CHORI	superpath	0.246	0.6295	0.6959	0.9163
PPGPPMET-	ppGpp bic	0.2441	0.6302	0.6985	0.9163
CHLOROPHY	chlorophy	0.2407	0.1015	0.01775	0.3815
PYRIDNUCS	NAD salva	0.2404	0.2996	0.4223	0.8498
P261-PWY	coenzyme	0.24	0.1183	0.04243	0.6001
PWY-5505	L-glutama	0.2385	0.2851	0.4027	0.8498

P108-PWY	pyruvate	0.2326	0.304	0.4443	0.8498
PWY-6737	starch de	0.2225	0.2683	0.4068	0.8498
PANTOSYN-	pantothen	0.2201	0.3062	0.4723	0.8498
PWY-6147	6-hydroxy	0.2156	0.3262	0.5087	0.8498
PWY-7539	6-hydroxy	0.2126	0.3258	0.514	0.8498
GLYCOLYSI	superpath	0.2125	0.3294	0.5188	0.8498
COA-PWY	coenzyme	0.2102	0.2795	0.452	0.8498
PWY-6163	chorismat	0.2091	0.2748	0.4467	0.8498
PANTO-PWY	phosphopa	0.209	0.3169	0.5095	0.8498
PWY-6895	superpath	0.2086	0.361	0.5633	0.8498
PWY-5529	superpath	0.2064	0.1493	0.1669	0.8498
RIBOSYN2-	flavin bi	0.2014	0.2988	0.5003	0.8498
PWY-5104	L-isoleuc	0.2006	0.2575	0.4358	0.8498
FERMENTAT	mixed aci	0.2003	0.2593	0.4397	0.8498
PWY-5695	urate bio	0.1999	0.3194	0.5313	0.8498
NONMEVIPP	methylery	0.1997	0.2773	0.4714	0.8498
PWY-7560	methylery	0.1997	0.2773	0.4714	0.8498
PWY-6123	inosine-5	0.1989	0.2849	0.485	0.8498
1CMET2-PW	N10-formy	0.1984	0.2733	0.468	0.8498
PWY-5101	L-isoleuc	0.1959	0.2517	0.4364	0.8498
GLYCOGENS	glycogen	0.1958	0.2621	0.4551	0.8498
ILEUSYN-P	L-isoleuc	0.195	0.2549	0.4442	0.8498
VALSYN-PW	L-valine	0.195	0.2549	0.4442	0.8498
PWY-5121	superpath	0.1944	0.277	0.4826	0.8498
ASPASN-PW	superpath	0.1938	0.2919	0.5068	0.8498
PWY-6612	superpath	0.1907	0.32	0.5514	0.8498
PWY-2942	L-lysine	0.1901	0.2728	0.4858	0.8498
GLUTORN-P	L-ornithi	0.19	0.2877	0.5089	0.8498
PWY-5686	UMP biosy	0.1892	0.2734	0.4889	0.8498
FOLSYN-PW	superpath	0.1867	0.3003	0.5341	0.8498
PWY-6892	thiazole	0.1865	0.2834	0.5105	0.8498
ARO-PWY	chorismat	0.1857	0.2663	0.4857	0.8498
PWY-5103	L-isoleuc	0.1852	0.2473	0.4541	0.8498
PWY-7219	adenosine	0.185	0.2729	0.498	0.8498
PWY-7208	superpath	0.1833	0.2676	0.4933	0.8498
PWY-7347	sucrose b	0.1815	0.8616	0.8332	0.9413
COMPLETE-	superpath	0.1805	0.2661	0.4974	0.8498
P441-PWY	superpath	0.179	0.2982	0.5483	0.8498
PWY-7199	pyrimidin	0.1779	0.3093	0.5652	0.8498
ANAGLYCOL	glycolysi	0.1777	0.2697	0.51	0.8498
HISTSYN-P	L-histidi	0.1772	0.2568	0.4902	0.8498
PWY-5097	L-lysine	0.1753	0.2709	0.5175	0.8498
TRNA-CHAR	tRNA char	0.1744	0.2724	0.522	0.8498
PWY-6121	5-aminoim	0.1724	0.2657	0.5163	0.8498
PWY-5088	L-glutama	0.1721	0.668	0.7967	0.9413
PWY-6126	superpath	0.1719	0.2758	0.5331	0.8498
DTDPRHAMS	dTDP-L-rh	0.1713	0.2843	0.547	0.8498
PEPTIDOGL	peptidogl	0.1712	0.2727	0.5301	0.8498
PWY-6122	5-aminoim	0.1711	0.2671	0.5218	0.8498
PWY-6277	superpath	0.1711	0.2671	0.5218	0.8498
BRANCHED-	superpath	0.1698	0.2475	0.4926	0.8498

PWY-6385	peptidogl	0.1698	0.273	0.534	0.8498
PWY-6387	UDP-N-ace	0.1698	0.2723	0.5329	0.8498
PWY-6386	UDP-N-ace	0.1695	0.2726	0.5342	0.8498
TCA	TCA cycle	0.1693	0.3181	0.5946	0.8606
PWY-7229	superpath	0.1687	0.2756	0.5406	0.8498
PWY-3001	superpath	0.1643	0.2435	0.4999	0.8498
PWY-6897	thiamin s	0.1627	0.283	0.5654	0.8498
THRESYN-F	superpath	0.1626	0.2434	0.504	0.8498
PWY-6608	guanosine	0.1617	0.2975	0.5866	0.8567
PWY-7111	pyruvate	0.1608	0.2777	0.5626	0.8498
PWY0-1338	polymyxin	0.16	1.05	0.8789	0.9657
NONOXIPEN	pentose p	0.1583	0.2711	0.5591	0.8498
PWY-5667	CDP-diacy	0.1568	0.2771	0.5714	0.8498
PWY0-1319	CDP-diacy	0.1568	0.2771	0.5714	0.8498
PWY-5100	pyruvate	0.1564	0.2728	0.5663	0.8498
PWY0-1061	superpath	0.1545	0.7047	0.8265	0.9413
DAPLYSINE	L-lysine	0.1532	0.2789	0.5827	0.8542
METH-ACET	methanoge	0.1521	0.5667	0.7884	0.9413
PWY-7221	guanosine	0.1507	0.2711	0.5782	0.8508
SALVADEHY	adenosine	0.1491	0.2995	0.6185	0.8704
PWY-6353	purine nu	0.1448	0.268	0.589	0.857
PWY0-162	superpath	0.1424	0.3937	0.7176	0.9257
GLYCOCAT-	glycogen	0.1412	0.2695	0.6003	0.8606
PWY-841	superpath	0.141	0.3741	0.7062	0.9163
PWY-7196	superpath	0.1406	0.4399	0.7492	0.9366
PWY-6545	pyrimidin	0.1405	0.3294	0.6698	0.9064
PWY-7220	adenosine	0.136	0.2992	0.6495	0.8928
PWY-7222	guanosine	0.136	0.2992	0.6495	0.8928
PWY0-41	allantoin	0.1353	0.9786	0.8901	0.973
UBISYN-PW	superpath	0.1284	0.558	0.818	0.9413
PHOSLIPSY	superpath	0.1276	0.2817	0.6506	0.8928
PYRIDOXSY	pyridoxal	0.1274	0.5856	0.8278	0.9413
ARGSYNBSU	L-argininr	0.1263	0.2919	0.6652	0.9033
METHYLGAL	methylgal	0.1262	0.09744	0.1951	0.8498
PWY-5855	ubiquinol	0.1253	0.5543	0.8211	0.9413
PWY-5856	ubiquinol	0.1253	0.5543	0.8211	0.9413
PWY-5857	ubiquinol	0.1253	0.5543	0.8211	0.9413
PWY-6708	ubiquinol	0.1253	0.5543	0.8211	0.9413
PWY-6125	superpath	0.1229	0.4098	0.7642	0.9413
PWY-6728	methylasp	0.1205	0.5991	0.8405	0.9413
PWY0-845	superpath	0.1199	0.5765	0.8352	0.9413
PWY-7228	superpath	0.1195	0.4264	0.7793	0.9413
PWY-7400	L-argininr	0.1135	0.2873	0.6929	0.9163
ARGSYN-PW	L-argininr	0.1129	0.2882	0.6951	0.9163
DENOVOPUR	superpath	0.1128	0.3624	0.7555	0.9402
PWY-5659	GDP-mannc	0.1115	0.2962	0.7066	0.9163
PWY-6151	S-adenosy	0.1089	0.2909	0.708	0.9163
P124-PWY	Bifidobac	0.1049	0.4452	0.8137	0.9413
GALLATE-D	gallate d	0.1042	0.08108	0.1988	0.8498
PWY-5188	tetrapyrr	0.1027	0.2945	0.7273	0.932
P184-PWY	protocate	0.1021	0.06357	0.1081	0.8498

PWY-1361	benzoyl-C	0.09993	0.06463	0.1221	0.8498
PWY4FS-7	phosphati	0.09965	0.2972	0.7374	0.9326
PWY4FS-8	phosphati	0.09965	0.2972	0.7374	0.9326
PWY-5860	superpath	0.09688	1.073	0.928	0.9842
PWY-5862	superpath	0.09688	1.073	0.928	0.9842
GLYCOLYSI	glycolysi	0.0965	0.286	0.7358	0.9326
PWY-5845	superpath	0.09626	1.069	0.9283	0.9842
PWY-5850	superpath	0.09626	1.069	0.9283	0.9842
PWY-5896	superpath	0.09626	1.069	0.9283	0.9842
PWY-7200	superpath	0.09535	0.3439	0.7816	0.9413
PWY-7197	pyrimidin	0.08998	0.4305	0.8344	0.9413
PWY-6609	adenine a	0.08653	0.3054	0.7769	0.9413
PWY-7184	pyrimidin	0.07929	0.3702	0.8304	0.9413
P122-PWY	heterolac	0.0707	0.365	0.8464	0.9413
PWY0-166	superpath	0.07061	0.2988	0.8132	0.9413
PWY-6317	galactose	0.07	0.2692	0.7949	0.9413
PWY-5189	tetrapyrr	0.06992	0.3002	0.8158	0.9413
PWY-7187	pyrimidin	0.0671	0.2962	0.8208	0.9413
ANAEROFRU	homolacti	0.06427	0.2982	0.8294	0.9413
OANTIGEN-	O-antigen	0.04539	0.3028	0.8808	0.9657
PWY-5484	glycolysi	0.03752	0.3293	0.9093	0.9829
CRNFORCAT	creatinin	0.03406	0.1747	0.8455	0.9413
PWY-6581	spirillox	0.03147	0.02641	0.2334	0.8498
PWY0-1277	3-phenylp	0.0312	0.02799	0.2651	0.8498
POLYAMINS	superpath	0.02557	0.4764	0.9572	1
P281-PWY	3-phenylp	0.02232	0.9268	0.9808	1
PWY-5183	superpath	0.02071	0.06137	0.7357	0.9326
PWY-1422	vitamin E	0.01885	0.01606	0.2407	0.8498
SUCSYN-PW	sucrose b	0.01822	0.8511	0.9829	1
GALLATE-D	gallate d	0.01573	0.07109	0.8249	0.9413
VALDEG-PW	L-valine	0.01343	0.03347	0.6881	0.9163
PWY-6713	L-rhamnos	0.009946	0.01165	0.3931	0.8498
PWY-5198	factor 42	0.008837	0.02202	0.6882	0.9163
HCAMHPDEG	3-phenylp	0.008317	0.007465	0.2652	0.8498
PWY-6690	cinnamate	0.008317	0.007465	0.2652	0.8498
AEROBACTI	aerobacti	0.005019	0.003948	0.2037	0.8498
P164-PWY	purine nu	0.003879	0.415	0.9925	1
GLCMANNAN	superpath	0.002679	0.3278	0.9935	1
PWY0-1298	superpath	0.00081	0.3318	0.9981	1
P621-PWY	nylon-6 c	0	0.000155	1	1
PWY-5532	adenosine	0	0.000155	1	1
PWY-5941	glycogen	0	0.000155	1	1
PWY-6165	chorismat	0	0.000155	1	1
PWY-6174	mevalonat	0	0.000155	1	1
PWY-6338	superpath	0	0.000155	1	1
PWY-6565	superpath	0	0.000155	1	1
PWY-6731	starch de	0	0.000155	1	1
PWY-6992	1,5-anhyd	0	0.000155	1	1
PWY-7097	vanillin	0	0.000155	1	1
PWY-7098	vanillin	0	0.000155	1	1
PWY-7185	UTP and C	0	0.000155	1	1

PWY-7391	isoprene	0	0.000155	1	1
PWY-6107	chlorosal	-0.00208	0.97	0.9983	1
PWY-7031	protein N	-0.00806	0.0306	0.7923	0.9413
ARGDEG-PW	superpath	-0.01179	0.09892	0.9051	0.9812
ORNARGDEG	superpath	-0.01179	0.09892	0.9051	0.9812
PWY-6948	sitosterc	-0.01735	0.02281	0.4468	0.8498
PWY-7255	ergothion	-0.02426	0.04651	0.602	0.8606
PWY0-1297	superpath	-0.02775	0.3549	0.9377	0.9888
PWY0-1296	purine ri	-0.03099	0.3662	0.9325	0.9861
UDPNAGSYN	UDP-N-ace	-0.03657	0.3915	0.9256	0.9842
PWY-621	sucrose c	-0.04837	0.3223	0.8807	0.9657
PWY-6891	thiazole	-0.04901	0.5353	0.927	0.9842
PWY-6901	superpath	-0.05678	0.2844	0.8417	0.9413
P161-PWY	acetylene	-0.0702	0.3765	0.8521	0.9449
ARG+POLYA	superpath	-0.07036	0.3961	0.859	0.9498
RHAMCAT-F	L-rhamnos	-0.08677	0.3142	0.7824	0.9413
PWY-181	photoresp	-0.09026	0.6718	0.8931	0.9736
PENTOSE-F	pentose p	-0.09543	0.3464	0.783	0.9413
P23-PWY	reductive	-0.09596	0.2973	0.7469	0.9366
PWY-7431	aromatic	-0.1016	0.5058	0.8408	0.9413
LIPASYN-F	phospholi	-0.1063	0.1184	0.3693	0.8498
PWY-3661	glycine b	-0.1092	0.1796	0.5429	0.8498
PWY-7456	mannan de	-0.1112	0.3484	0.7497	0.9366
PWY-5918	superpath	-0.117	0.3028	0.6993	0.9163
PWY-5913	TCA cycle	-0.118	0.4007	0.7684	0.9413
PWY0-1415	superpath	-0.1241	0.4956	0.8023	0.9413
HEME-BIOS	heme bios	-0.1638	0.3398	0.6299	0.8769
PWY-6404	superpath	-0.1643	0.216	0.4468	0.8498
PWY-7211	superpath	-0.1651	0.4299	0.701	0.9163
PWY-1541	superpath	-0.1717	0.8626	0.8422	0.9413
PWY-5347	superpath	-0.1833	0.2979	0.5383	0.8498
PWY-7003	glycerol	-0.1895	0.7808	0.8083	0.9413
ARGORNPRC	arginine,	-0.1905	0.5986	0.7502	0.9366
HSERMETAN	L-methion	-0.2091	0.3555	0.5565	0.8498
PWY-5384	sucrose c	-0.2341	0.658	0.7221	0.9284
MET-SAM-F	superpath	-0.2378	0.3235	0.4623	0.8498
POLYAMSYN	superpath	-0.2531	0.499	0.612	0.8676
HOMOSER-M	L-methion	-0.2589	0.3369	0.4422	0.8498
PWY0-1261	anhydromu	-0.2726	0.4731	0.5646	0.8498
CODH-PWY	reductive	-0.294	0.6413	0.6466	0.8928
PWY-7295	L-arabinc	-0.3071	0.2108	0.1452	0.8498
DENITRIFI	nitrate r	-0.3076	0.5976	0.6067	0.8632
HEXITOLDE	superpath	-0.3218	0.4496	0.4741	0.8498
PWY-5022	4-aminobu	-0.3384	0.7021	0.6299	0.8769
PWY0-781	aspartate	-0.3449	0.3812	0.3656	0.8498
PWY-5180	toluene c	-0.3475	0.781	0.6564	0.8945
PWY-5182	toluene c	-0.3475	0.781	0.6564	0.8945
TEICHOICA	teichoic	-0.3485	0.4304	0.4181	0.8498
P4-PWY	superpath	-0.3525	0.3735	0.3452	0.8498
ENTBACSYN	enterobac	-0.3782	0.7517	0.6149	0.8685
THREOCAT-	superpath	-0.3788	0.7188	0.5982	0.8606

PWY-7377	cob(II)yr	-0.4135	0.737	0.5747	0.8498
PRPP-PWY	superpath	-0.4349	0.4018	0.279	0.8498
PWY-4984	urea cycl	-0.4503	0.5893	0.4448	0.8498
PWY-5415	catechol	-0.4887	0.9387	0.6026	0.8606
PWY-5005	biotin bi	-0.5002	0.8929	0.5753	0.8498
PWY0-1479	tRNA proc	-0.5257	0.4702	0.2635	0.8498
PWY-6630	superpath	-0.5348	0.6143	0.384	0.8498
PWY-6628	superpath	-0.5489	0.6186	0.3749	0.8498
PWY-6071	superpath	-0.5599	0.6298	0.3741	0.8498
PWY-3781	aerobic r	-0.5604	0.3177	0.07771	0.8354
HEMESYN2-	heme bios	-0.563	0.327	0.08513	0.8498
PWY-2941	L-lysine	-0.5832	0.6498	0.3695	0.8498
P125-PWY	superpath	-0.5882	0.7764	0.4487	0.8498
P461-PWY	hexitol f	-0.5934	0.5553	0.2852	0.8498
RUMP-PWY	formaldeh	-0.6047	0.5835	0.3001	0.8498
PWY-5747	2-methylc	-0.6091	0.5715	0.2865	0.8498
GLYCOLYSI	superpath	-0.6141	0.4254	0.1488	0.8498
GLYOXYLAT	glyoxylat	-0.6288	0.5377	0.2422	0.8498
GLUCOSE1P	glucose a	-0.6412	0.655	0.3276	0.8498
PWY-5920	superpath	-0.6659	0.622	0.2844	0.8498
PWY-5345	superpath	-0.6661	0.3711	0.07268	0.8273
PWY-1861	formaldeh	-0.6907	0.6768	0.3074	0.8498
TCA-GLYOX	superpath	-0.7041	0.4543	0.1212	0.8498
PWY0-42	2-methylc	-0.7084	0.5598	0.2058	0.8498
PWY-6397	mycolyl-a	-0.7121	1.04	0.4935	0.8498
PWY-7392	taxadiene	-0.7795	0.6187	0.2077	0.8498
AST-PWY	L-argininr	-0.7824	0.6924	0.2585	0.8498
PWY-5507	adenosylc	-0.8311	0.8048	0.3017	0.8498
PWY-6396	superpath	-0.8385	0.9918	0.3979	0.8498
PWY-7527	L-methion	-0.8794	0.938	0.3485	0.8498
PWY-4361	S-methyl-	-0.9004	0.9619	0.3492	0.8498
P105-PWY	TCA cycle	-0.936	0.8219	0.2548	0.8498
P381-PWY	adenosylc	-0.9504	0.758	0.2099	0.8498
P101-PWY	ectoine b	-0.9583	0.9638	0.3201	0.8498
PWY490-3	nitrate r	-0.962	0.8065	0.233	0.8498
PWY-7376	cob(II)yr	-0.963	0.7652	0.2082	0.8498
PWY-7234	inosine-5	-0.9656	0.8259	0.2423	0.8498
PWY-5417	catechol	-0.9901	0.7736	0.2006	0.8498
PWY-5431	aromatic	-0.9901	0.7736	0.2006	0.8498
PWY-5178	toluene d	-0.9919	NaN	NaN	NaN
PWY-5838	superpath	-1.01	1.022	0.323	0.8498
PWY-6906	chitin de	-1.018	0.7487	0.1739	0.8498
PWY-5840	superpath	-1.021	1.027	0.3203	0.8498
LEU-DEG2-	L-leucine	-1.025	0.7299	0.1601	0.8498
PWY-5861	superpath	-1.031	1.034	0.3187	0.8498
PWY-5897	superpath	-1.032	1.033	0.318	0.8498
PWY-5898	superpath	-1.032	1.033	0.318	0.8498
PWY-5899	superpath	-1.032	1.033	0.318	0.8498
PWY-6182	superpath	-1.065	0.631	0.09147	0.8498
PWY0-321	phenylace	-1.08	0.555	0.05177	0.6401
PWY-5863	superpath	-1.098	1.072	0.3058	0.8498

PWY-5837	1,4-dihyd	-1.1	1.073	0.3054	0.8498
PWY-7094	fatty aci	-1.124	0.7127	0.1146	0.8498
PWY-5910	superpath	-1.178	0.9935	0.2356	0.8498
PWY-922	mevalonat	-1.186	1.001	0.236	0.8498
PWY-722	nicotinat	-1.301	1.02	0.2022	0.8498
PROTOCATE	protocate	-1.328	1.063	0.2117	0.8498
TYRFUMCAT	L-tyrosin	-1.351	0.4857	0.005393	0.1897
PWY-5420	catechol	-1.368	1.125	0.2241	0.8498
CATECHOL-	catechol	-1.381	1.104	0.2108	0.8498
PWY-5419	catechol	-1.483	1.202	0.2171	0.8498
PWY-6470	peptidogl	-1.486	1.125	0.1866	0.8498
P221-PWY	octane ox	-1.55	0.968	0.1093	0.8498
PWY-6210	2-aminoph	-1.556	1.004	0.1212	0.8498
PWY-6562	norspermi	-1.594	0.5936	0.007241	0.2
PWY-5655	L-tryptop	-1.629	0.8729	0.06198	0.7269
PWY-5028	L-histidi	-1.682	0.3994	2.55E-05	0.009858
GOLPDLCAT	superpath	-1.777	NaN	NaN	NaN
PWY-5181	toluene d	-1.825	0.576	0.001531	0.1173
PWY-6185	4-methylc	-1.858	0.6136	0.002455	0.1188
PWY-6876	isopropan	-1.903	1.349	0.1584	0.8498
DHGLUCONA	glucose d	-2.029	0.8878	0.02231	0.4317
PWY-5654	2-amino-3	-2.065	0.7241	0.004337	0.1678
LACTOSECA	lactose a	-2.077	0.9399	0.02712	0.4771
PWY-7616	methanol	-2.167	0.5769	0.000172	0.02222
PWY-5647	2-nitrobe	-2.173	0.7893	0.005894	0.1901
PWY-5265	peptidogl	-2.207	0.9468	0.01973	0.4019
NADSYN-PW	NAD biosy	-2.271	0.739	0.00212	0.1173
PWY-5651	L-tryptop	-2.273	0.7398	0.002122	0.1173
PWY-6505	L-tryptop	-2.299	0.7995	0.004031	0.1678
PWY-5430	meta clea	-2.369	0.6573	0.000313	0.03025
PWY-6383	mono-tran	-2.629	1.023	0.01017	0.2315
PWY1G-0	mycothiol	-2.66	1.031	0.009894	0.2315
PWY-7007	methyl ke	-3.268	0.8135	5.88E-05	0.01137

phylum

ID	M1	M2	M3	M4	M5	M6	M8	K1
Bacteroid	0.264676	0.171506	0.483209	0.067191	0.663354	0.432761	0.606864	0.837383
Firmicute	0.501191	0.598783	0.411019	0.824274	0.310895	0.504274	0.327246	0.146686
Actinobac	0.212254	0.208607	0.054489	0.097563	0.005951	0.02872	0.03577	0.000445
Proteobac	0.008611	0.009846	0.034942	0.004699	0.009158	0.019914	0.017454	0.006377
Tenericut	0.001596	0.000804	0.006629	0.000796	0.001224	0.001381	0.000445	0.000127
Elusimicr	0.000314	0.000913	0.00102	0	0.000779	0.002245	0.000139	0.000508
Cyanobact	0.000105	0.000435	0.001505	0.000136	0.000371	0.000288	0.001615	0.002224
TM7	0.000157	0.002304	0.000146	0.001476	0.000148	0.000345	0.000139	0
Spirochae	0	0	0	0	0.000204	8.63E-05	5.57E-05	0
Deferriba	0	0	4.86E-05	5.83E-05	0	0	0	8.47E-05
Others	0.011097	0.006803	0.006993	0.003806	0.007916	0.009986	0.010272	0.006165

K2	K3	K4	K5	K6	K7	DX1	DX2	DX3
0.562139	0.809646	0.841511	0.583007	0.419274	0.213321	0.789078	0.716719	0.63663
0.412749	0.173867	0.144293	0.391778	0.544893	0.75139	0.182471	0.259771	0.343202
0.00054	0.000744	0.00034	0.001022	0.002876	0.00226	0.001468	0.008845	0.004273
0.009698	0.007974	0.005716	0.011735	0.023125	0.022931	0.016995	0.005843	0.01159
0.007336	7.69E-05	4.25E-05	0.003364	0.002389	0.001818	0.000133	0.000159	0.000353
0.00189	0.000462	0.000723	0.000972	0.003786	0.002136	0.000133	0.001432	0.000269
0.00153	0.001103	0.001105	0.003314	0.000683	0.001642	0.000289	0.000523	0.000521
0.000743	0.000282	0	0.000174	0.000845	0.001095	0	0.000159	0.000353
0.000158	0	0	0	0	0.000918	8.90E-05	6.82E-05	8.41E-05
0	5.13E-05	0	7.48E-05	0	0.000194	0	0.000136	0
0.003218	0.005795	0.006269	0.00456	0.002129	0.002295	0.009343	0.006344	0.002725

DX4	DX5	DX6	DX7	G1	G3	G4	G5	G6
0.391609	0.4619	0.634294	0.559292	0.220357	0.420308	0.620673	0.659924	0.497115
0.577082	0.51053	0.331581	0.409689	0.753944	0.555591	0.36337	0.32826	0.479468
0.004062	0.004761	0.008784	0.006449	0.002822	0.000941	0.002391	0.000249	0.001785
0.015203	0.015708	0.016619	0.015856	0.017215	0.013896	0.005977	0.007093	0.005788
0.001169	0.000814	0.000268	0.000388	0.000586	0.000897	0.002572	0.00065	0.008514
0.004698	0.002747	0.000907	0.000388	0.000781	0.000159	0.001503	0.000134	0.002932
0.001477	0.000733	0.000557	0.001551	0.001473	0.006716	0.001612	0.001052	0.000526
0.000616	0.000244	0.000351	0.000327	0.001597	0.000492	0.000254	0.000249	0.000545
0.000164	0	0.000309	0.000122	0	0	0	0	0
8.21E-05	0	0	0	3.55E-05	0	5.43E-05	0	7.52E-05
0.003837	0.002564	0.00633	0.005938	0.001189	0.000999	0.001594	0.00239	0.003251

G7	G8	Z1	Z2	Z3	Z4	Z5	Z6	Z7
0.411646	0.452242	0.210225	0.630139	0.769513	0.427727	0.359414	0.275222	0.387636
0.567602	0.507427	0.75925	0.35391	0.216358	0.558682	0.606222	0.683792	0.601902
0.003245	0.004416	0.004332	0.001791	0.000482	0.000553	0.01174	0.000725	0.000949
0.009218	0.029183	0.019132	0.007204	0.008624	0.009295	0.015286	0.030637	0.004167
0.002596	0.002402	0.001354	0.00021	0.00039	0.000957	0.000367	0.001156	0.000703
0.000973	0.000508	0.001827	0.002497	0.000528	0.000255	0.002519	0.000804	0.00116
0.000708	0.000406	0.00018	0.000648	0.001307	0.000468	0.000122	0.002097	0.000826
0.001224	0.001489	0.000835	0.000114	0.000138	0.000468	0.000587	0.000686	0.001037
0.000206	0	0.000158	5.72E-05	4.59E-05	0	0	0.001764	0
0	0	6.77E-05	0	0	0	0	0.001215	0
0.002581	0.001929	0.00264	0.00343	0.002615	0.001595	0.003742	0.001901	0.001618

D1	D2	D3	D4	D5	D6	D7
0.735779	0.592705	0.551915	0.199381	0.563919	0.586654	0.243422
0.241535	0.351887	0.421368	0.762619	0.404931	0.385629	0.720489
0.003368	0.020551	0.003739	0.001487	0.002846	0.000448	0.010179
0.006712	0.021289	0.015163	0.014657	0.021951	0.013032	0.015565
0.004164	0.008008	0.001168	0.008469	0.002091	0	0.002729
0.001115	0.000936	0.00013	0.007497	0.000692	0.007987	0.00083
0.001274	0.001206	0.001039	0.001091	0.00206	0.002746	0.000593
0.00025	0.000828	0.001013	0.001765	0.000142	0.000252	0.000522
6.83E-05	0	0.000182	0.000317	0	0	0.000166
0	7.20E-05	0	0.000337	0.000236	0	7.12E-05
0.005734	0.002519	0.004284	0.00238	0.001132	0.003251	0.005434

GABA

	1	2	3	4	5	6	7					
A	0.03	0.37	0.275	0.293	0.331	0.399	0.402					450
B	0.586	0.381	0.269	0.328	0.351	0.394	0.393					450
C	0.361	0.355	0.275	0.283	0.294	0.369	0.385					450
D	0.22	0.358	0.303	0.319	0.322	0.394	0.433					450
E	0.166	0.365	0.28	0.312	0.34	0.411	0.468					450
F	0.143	0.369	0.282	0.354	0.352	0.404	0.385					450
G		0.358	0.3	0.344	0.32	0.375	0.377					450
H		0.358	0.296	0.34	0.345	0.358	0.384					450

	1	2	3	4	5	6	7
A	0	0.34	0.245	0.263	0.301	0.369	0.372
B	0.556	0.351	0.239	0.298	0.321	0.364	0.363
C	0.331	0.325	0.245	0.253	0.264	0.339	0.355
D	0.19	0.328	0.273	0.289	0.292	0.364	0.403
E	0.136	0.335	0.25	0.282	0.31	0.381	0.438
F	0.113	0.339	0.252	0.324	0.322	0.374	0.355
G		0.328	0.27	0.314	0.29	0.345	0.347
H		0.328	0.266	0.31	0.315	0.328	0.354

	u mol/L
0.556	32
0.331	16
0.19	8
0.136	4
0.113	2

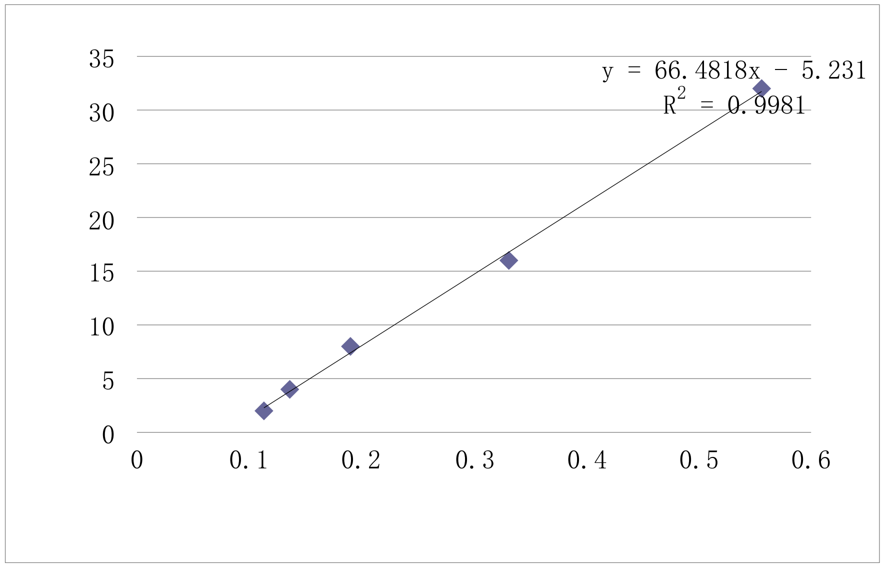

Concentrations	K	M	DX	D	Z	G
A	17.37	11.06	12.25	14.78	19.30	19.50
B	18.10	10.66	14.58	16.11	18.97	18.90
C	16.38	11.06	11.59	12.32	17.31	18.37
D	16.57	12.92	13.98	14.18	18.97	21.56
E	17.04	11.39	13.52	15.38	20.10	23.89
F	17.31	11.52	16.31	16.18	19.63	18.37
G	16.57	12.72	15.64	14.05	17.70	17.84
H	16.57	12.45	15.38	15.71	16.57	18.30

	K	M	DX	D	Z	G
mean value	16.99	11.72	14.16	14.84	18.57	19.59

standard d	0.55	0.80	1.55	1.22	1.15	1.95

GAD65

	1	2	3	4	6	7	8					
A	0.032	0.438	0.354	0.424	0.372	0.405	0.45					450
B	0.567	0.414	0.328	0.419	0.343	0.393	0.426					450
C	0.417	0.446	0.366	0.384	0.35	0.38	0.408					450
D	0.225	0.445	0.363	0.411	0.374	0.392	0.443					450
E	0.161	0.429	0.376	0.429	0.297	0.393	0.436					450
F	0.121	0.448	0.387	0.419	0.323	0.405	0.438					450
G		0.426	0.377	0.453	0.342	0.399	0.421					450
H		0.419	0.36	0.514	0.362	0.396	0.422					450

	1	2	3	4	6	7	8
A	0	0.406	0.322	0.392	0.34	0.373	0.418
B	0.535	0.382	0.296	0.387	0.311	0.361	0.394
C	0.385	0.414	0.334	0.352	0.318	0.348	0.376
D	0.193	0.413	0.331	0.379	0.342	0.36	0.411
E	0.129	0.397	0.344	0.397	0.265	0.361	0.404
F	0.089	0.416	0.355	0.387	0.291	0.373	0.406
G		0.394	0.345	0.421	0.31	0.367	0.389
H		0.387	0.328	0.482	0.33	0.364	0.39

	ng/ml
0.535	2
0.385	1
0.193	0.5
0.129	0.25
0.089	0.125

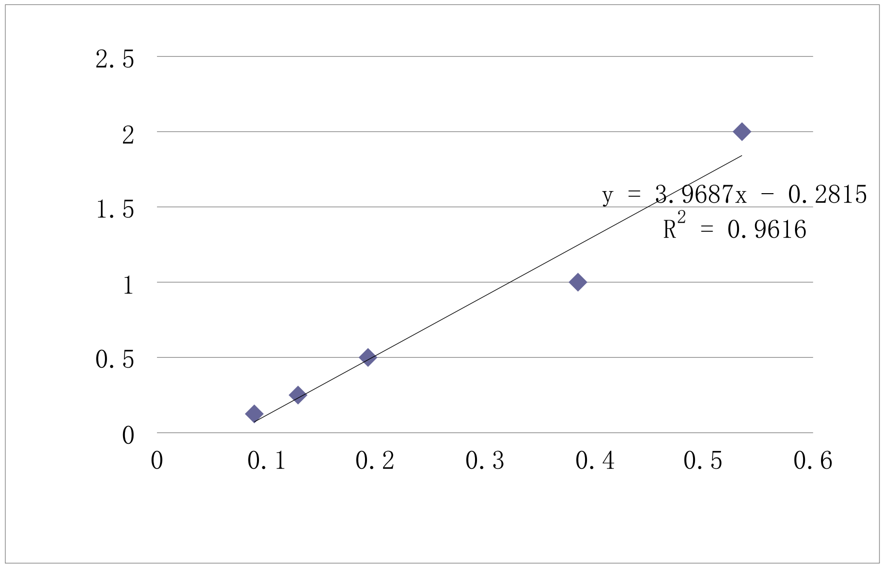

Concentrations	1	2	3	4	6	7	8
A		1.33	1.00	1.27	1.07	1.20	1.38
B		1.23	0.89	1.25	0.95	1.15	1.28
C		1.36	1.04	1.12	0.98	1.10	1.21
D		1.36	1.03	1.22	1.08	1.15	1.35
E		1.29	1.08	1.29	0.77	1.15	1.32
F		1.37	1.13	1.25	0.87	1.20	1.33
G		1.28	1.09	1.39	0.95	1.18	1.26
H		1.25	1.02	1.63	1.03	1.16	1.27

	K	M	DX	D	Z	G
mean value	1.31	1.04	1.30	0.96	1.16	1.30

standard d	0.05	0.07	0.14	0.10	0.03	0.05

Gln

	1	2	3	4	5	6	7					
A	0.03	0.37	0.276	0.288	0.35	0.408	0.457					450
B	0.504	0.364	0.261	0.284	0.41	0.431	0.415					450
C	0.323	0.354	0.254	0.288	0.304	0.36	0.349					450
D	0.201	0.375	0.296	0.297	0.329	0.39	0.384					450
E	0.146	0.369	0.274	0.313	0.328	0.407	0.394					450
F	0.108	0.357	0.282	0.313	0.347	0.377	0.372					450
G		0.351	0.275	0.311	0.316	0.367	0.42					450
H		0.351	0.275	0.308	0.324	0.362	0.397					450

	1	2	3	4	5	6	7
A	0	0.34	0.246	0.258	0.32	0.378	0.427
B	0.474	0.334	0.231	0.254	0.38	0.401	0.385
C	0.293	0.324	0.224	0.258	0.274	0.33	0.319
D	0.171	0.345	0.266	0.267	0.299	0.36	0.354
E	0.116	0.339	0.244	0.283	0.298	0.377	0.364
F	0.078	0.327	0.252	0.283	0.317	0.347	0.342
G		0.321	0.245	0.281	0.286	0.337	0.39
H		0.321	0.245	0.278	0.294	0.332	0.367

	u mol/L
0.474	600
0.293	300
0.171	150
0.116	75
0.078	37.5

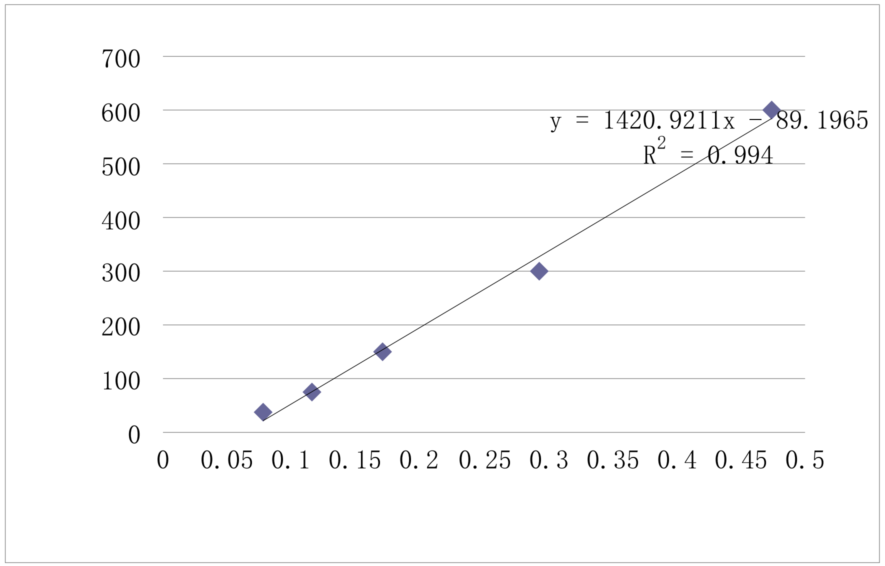

Concentrations	1	2	3	4	5	6	7
A		393.61	260.13	277.17	365.21	447.57	517.15
B		385.09	238.83	271.49	450.41	480.23	457.51
C		370.89	228.89	277.17	299.89	379.41	363.79
D		400.71	288.53	289.95	335.39	422.01	413.49
E		392.19	257.29	312.67	333.97	446.15	427.69
F		375.15	268.65	312.67	360.95	403.55	396.45
G		366.63	258.71	309.83	316.93	389.35	464.61
H		366.63	258.71	305.57	328.29	382.25	431.95

	K	M	DX	D	Z	G
mean value	381.36	257.47	294.57	348.88	418.82	434.08

standard d	12.44	16.82	16.46	43.26	34.18	43.60

Glu

	1	2	3	4	5	6	7					
A	0.029	0.334	0.518	0.458	0.449	0.445	0.423					450
B	0.536	0.296	0.428	0.333	0.442	0.364	0.375					450
C	0.333	0.282	0.471	0.41	0.455	0.425	0.419					450
D	0.185	0.276	0.461	0.42	0.461	0.418	0.386					450
E	0.153	0.329	0.5	0.467	0.502	0.481	0.445					450
F	0.13	0.34	0.483	0.465	0.491	0.452	0.434					450
G		0.32	0.498	0.453	0.481	0.447	0.443					450
H		0.324	0.512	0.49	0.479	0.438	0.459					450

	1	2	3	4	5	6	7
A	0	0.305	0.489	0.429	0.42	0.416	0.394
B	0.507	0.267	0.399	0.304	0.413	0.335	0.346
C	0.304	0.253	0.442	0.381	0.426	0.396	0.39
D	0.156	0.247	0.432	0.391	0.432	0.389	0.357
E	0.124	0.3	0.471	0.438	0.473	0.452	0.416
F	0.101	0.311	0.454	0.436	0.462	0.423	0.405
G	-0.029	0.291	0.469	0.424	0.452	0.418	0.414
H	-0.029	0.295	0.483	0.461	0.45	0.409	0.43

	u mol/L
0.507	12
0.304	6
0.156	3
0.124	1.5
0.101	0.75

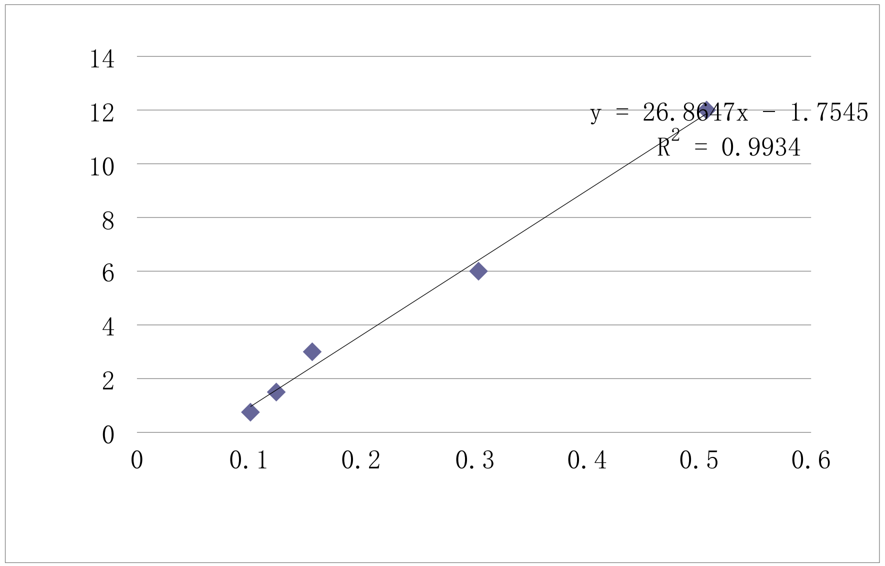

Concentrations	1	2	3	4	5	6	7
A		6.44	11.38	9.77	9.53	9.42	8.83
B		5.42	8.96	6.41	9.34	7.24	7.54
C		5.04	10.12	8.48	9.69	8.88	8.72
D		4.88	9.85	8.75	9.85	8.69	7.84
E		6.30	10.90	10.01	10.95	10.39	9.42
F		6.60	10.44	9.96	10.66	9.61	9.12
G		6.06	10.84	9.63	10.39	9.47	9.37
H		6.17	11.22	10.63	10.33	9.23	9.80

	K	M	DX	D	Z	G
mean value	5.86	10.46	9.20	10.09	9.12	8.83

standard d	0.62	0.75	1.24	0.54	0.85	0.73

GS

	1	2	3	4	5	6	7					
A	0.031	0.37	0.365	0.395	0.334	0.403	0.379					450
B	0.451	0.407	0.333	0.409	0.36	0.364	0.393					450
C	0.324	0.404	0.326	0.373	0.327	0.38	0.359					450
D	0.179	0.415	0.335	0.388	0.345	0.394	0.42					450
E	0.151	0.428	0.367	0.414	0.396	0.406	0.446					450
F	0.124	0.342	0.369	0.404	0.364	0.375	0.422					450
G		0.407	0.335	0.392	0.347	0.395	0.43					450
H		0.414	0.34	0.382	0.348	0.402	0.372					450

	1	2	3	4	5	6	7
A	0	0.339	0.334	0.364	0.303	0.372	0.348
B	0.42	0.376	0.302	0.378	0.329	0.333	0.362
C	0.293	0.373	0.295	0.342	0.296	0.349	0.328
D	0.148	0.384	0.304	0.357	0.314	0.363	0.389
E	0.12	0.397	0.336	0.383	0.365	0.375	0.415
F	0.093	0.311	0.338	0.373	0.333	0.344	0.391
G		0.376	0.304	0.361	0.316	0.364	0.399
H		0.383	0.309	0.351	0.317	0.371	0.341

	n mol/L
0.42	40
0.293	20
0.148	10
0.12	5
0.093	2.5

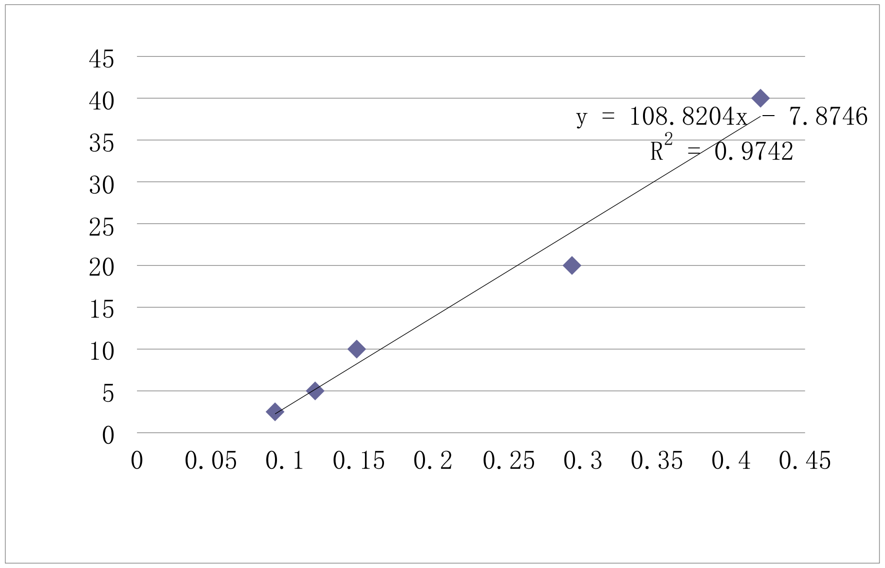

Concentrations	1	2	3	4	5	6	7
A		29.01	28.47	31.73	25.09	32.60	29.99
B		33.03	24.98	33.25	27.92	28.36	31.51
C		32.71	24.22	29.34	24.33	30.10	27.81
D		33.91	25.20	30.97	26.29	31.62	34.45
E		35.32	28.68	33.80	31.84	32.93	37.28
F		25.96	28.90	32.71	28.36	29.55	34.67
G		33.03	25.20	31.40	26.51	31.73	35.54
H		33.80	25.75	30.31	26.62	32.49	29.23

	K	M	DX	D	Z	G
mean value	32.10	26.43	31.69	27.12	31.17	32.56

standard d	2.87	1.79	1.41	2.17	1.54	3.17

Replicate	Group	Gabaar α 1 Ct (dRn)	Ct Gabra1正常	ΔCt Gabra1	β-actin Ct (dRn)	Ct β-actin正常	ΔCt β-actin	Efficiency	Relative Expression Ratio
1	K	26.24	26.67	0.43	27.56	27.85	0.29	E β-actin	**1.08**
2	K	26.9		-0.23	28.01		-0.16	103.5%	**0.96**
3	K	26.38		0.29	27.67		0.18	E Gabra1	**1.06**
4	K	26.91		-0.25	28.16		-0.31	95.8%	**1.06**
5	K	26.82		-0.16	28.04		-0.19		**1.03**
6	K	27.01		-0.35	27.95		-0.10		**0.85**
7	K	26.77		-0.11	27.88		-0.03		**0.95**
8	K	26.29		0.38	27.54		0.31		**1.03**
9	M	27.39		-0.73	27.91		-0.06		**0.64**
10	M	27.42		-0.76	27.92		-0.07		**0.63**
11	M	26.89		-0.23	27.53		0.32		**0.68**
12	M	27.66		-1.00	28.21		-0.36		**0.66**
13	M	28.09		-1.43	28.48		-0.63		**0.60**
14	M	27.72		-1.06	28.25		-0.40		**0.65**
15	M	27.02		-0.36	27.52		0.33		**0.62**
16	M	28.08		-1.42	28.4		-0.55		**0.57**
17	DX	27.88		-1.22	28.46		-0.61		**0.68**
18	DX	27.67		-1.01	28.34		-0.49		**0.72**
19	DX	27.51		-0.85	28.46		-0.61		**0.87**
20	DX	27.46		-0.80	28.13		-0.28		**0.71**
21	DX	27.37		-0.71	28.1		-0.25		**0.74**
22	DX	26.85		-0.19	27.77		0.08		**0.83**
23	DX	27.36		-0.70	28.18		-0.33		**0.79**
24	DX	27.12		-0.46	27.9		-0.05		**0.76**
25	D	27.16		-0.50	28.08		-0.23		**0.84**
26	D	27.24		-0.57	28.17		-0.32		**0.85**
27	D	27.64		-0.98	28.32		-0.47		**0.72**
28	D	27.4		-0.73	28.2		-0.35		**0.78**
29	D	26.86		-0.20	27.56		0.29		**0.71**
30	D	27.12		-0.46	27.84		0.01		**0.73**
31	D	27		-0.34	27.65		0.20		**0.69**
32	D	27.52		-0.86	28.12		-0.27		**0.68**
33	Z	27.51		-0.85	28.43		-0.58		**0.86**
34	Z	27.01		-0.35	27.99		-0.14		**0.88**
35	Z	27.5		-0.84	28.42		-0.57		**0.85**
36	Z	26.28		0.38	27.52		0.33		**1.02**
37	Z	27.39		-0.73	28.48		-0.63		**0.96**
38	Z	27.18		-0.52	28.18		-0.33		**0.89**
39	Z	26.34		0.32	27.58		0.27		**1.03**
40	G	26.81		-0.15	27.81		0.04		**0.88**
41	G	27.17		-0.51	28.35		-0.50		**1.02**
42	G	26.66		0.00	27.97		-0.12		**1.09**
43	G	26.41		0.25	27.66		0.19		**1.04**
44	G	26.59		0.07	27.94		-0.09		**1.12**
45	G	26.66		0.00	27.73		0.12		**0.92**

	mean valu	standard deviation
K	1.00	0.07
M	0.63	0.03
DX	0.77	0.06
D	0.75	0.06
Z	0.93	0.07
G	1.01	0.08

46 G	26.82	-0.16	27.97	-0.12		0.98
47 G	26.79	-0.13	28.02	-0.17		1.04

Replicate	Group	GluR-A Ct (dRn)	Ct_{GluR-A}正常	ΔCt_{GluR-A}	β-actin Ct (dRn)	$Ct_{\beta-actin}$正常	$\Delta Ct_{\beta-actin}$	Efficiency	Relative Expression Ratio
1	K	29.12	29.31	0.19	27.56	27.85	0.29	$E_{\beta-actin}$	**0.92**
2	K	29.41		-0.10	28.01		-0.16	103.5%	**1.05**
3	K	29.06		0.25	27.67		0.18	E_{GluR-A}	**1.04**
4	K	29.59		-0.28	28.16		-0.31	90.6%	**1.04**
5	K	29.4		-0.09	28.04		-0.19		**1.08**
6	K	29.6		-0.29	27.95		-0.10		**0.89**
7	K	29.44		-0.13	27.88		-0.03		**0.94**
8	K	28.89		0.42	27.54		0.31		**1.05**
9	M	27.66		1.65	27.91		-0.06		**3.03**
10	M	27.64		1.67	27.92		-0.07		**3.09**
11	M	26.97		2.34	27.53		0.32		**3.61**
12	M	27.85		1.46	28.21		-0.36		**3.32**
13	M	28.31		1.00	28.48		-0.63		**2.99**
14	M	27.93		1.38	28.25		-0.40		**3.24**
15	M	27.13		2.18	27.52		0.33		**3.23**
16	M	27.89		1.42	28.4		-0.55		**3.70**
17	DX	28.78		0.53	28.46		-0.61		**2.17**
18	DX	28.6		0.71	28.34		-0.49		**2.24**
19	DX	28.43		0.88	28.46		-0.61		**2.73**
20	DX	28.32		0.99	28.13		-0.28		**2.31**
21	DX	28.07		1.24	28.1		-0.25		**2.66**
22	DX	28.02		1.29	27.77		0.08		**2.17**
23	DX	28.48		0.83	28.18		-0.33		**2.16**
24	DX	27.72		1.59	27.9		-0.05		**2.89**
25	D	27.99		1.32	28.08		-0.23		**2.76**
26	D	28.33		0.98	28.17		-0.32		**2.37**
27	D	28.39		0.92	28.32		-0.47		**2.53**
28	D	28.22		1.09	28.2		-0.35		**2.59**
29	D	27.49		1.82	27.56		0.29		**2.64**
30	D	28.01		1.30	27.84		0.01		**2.30**
31	D	27.44		1.87	27.65		0.20		**2.90**
32	D	28.17		1.14	28.12		-0.27		**2.53**
33	Z	28.9		0.41	28.43		-0.58		**1.97**
34	Z	28.23		1.08	27.99		-0.14		**2.22**
35	Z	28.92		0.39	28.42		-0.57		**1.93**
36	Z	27.69		1.62	27.52		0.33		**2.25**
37	Z	28.9		0.41	28.48		-0.63		**2.04**
38	Z	28.43		0.88	28.18		-0.33		**2.23**
39	Z	27.73		1.58	27.58		0.27		**2.29**
40	G	28.15		1.16	27.81		0.04		**2.06**
41	G	28.85		0.46	28.35		-0.50		**1.92**
42	G	28.57		0.74	27.97		-0.12		**1.76**
43	G	28.04		1.27	27.66		0.19		**1.99**
44	G	28.36		0.95	27.94		-0.09		**1.97**
45	G	28.47		0.84	27.73		0.12		**1.58**

	mean valu	standard deviation
K	1.00	0.07
M	3.28	0.24
DX	2.42	0.28
D	2.58	0.18
Z	2.13	0.14
G	1.85	0.17

46 G	28.7	0.61	27.97	-0.12		1.62
47 G	28.51	0.80	28.02	-0.17		1.89

Replicate	Group	mGluR5<br>Ct (dRn)	$Ct_{mGluR5正常}$	ΔCt_{mGluR5}	β-actin<br>Ct (dRn)	$Ct_{β\text{-}actin正常}$	$\Delta Ct_{β\text{-}actin}$	Efficiency	Relative Expression Ratio
1	K	27.83	28.15	0.32	27.56	27.85	0.29	$E_{β\text{-}actin}$	1.02
2	K	28.28		-0.13	28.01		-0.16	103.5%	1.02
3	K	28.06		0.09	27.67		0.18	E_{mGluR5}	0.94
4	K	28.53		-0.38	28.16		-0.31	107.3%	0.94
5	K	28.14		0.01	28.04		-0.19		1.15
6	K	28.29		-0.14	27.95		-0.10		0.97
7	K	28.01		0.14	27.88		-0.03		1.13
8	K	28.03		0.12	27.54		0.31		0.87
9	M	27.38		0.77	27.91		-0.06		1.82
10	M	27.37		0.78	27.92		-0.07		1.85
11	M	26.68		1.47	27.53		0.32		2.32
12	M	27.44		0.71	28.21		-0.36		2.16
13	M	27.61		0.54	28.48		-0.63		2.31
14	M	27.68		0.47	28.25		-0.40		1.86
15	M	26.96		1.19	27.52		0.33		1.88
16	M	27.72		0.43	28.4		-0.55		2.02
17	DX	28.42		-0.27	28.46		-0.61		1.26
18	DX	28.24		-0.09	28.34		-0.49		1.32
19	DX	28.37		-0.22	28.46		-0.61		1.31
20	DX	28.04		0.11	28.13		-0.28		1.32
21	DX	27.94		0.21	28.1		-0.25		1.39
22	DX	27.54		0.61	27.77		0.08		1.47
23	DX	27.99		0.16	28.18		-0.33		1.42
24	DX	27.67		0.48	27.9		-0.05		1.46
25	D	27.99		0.16	28.08		-0.23		1.32
26	D	27.8		0.35	28.17		-0.32		1.61
27	D	28.06		0.09	28.32		-0.47		1.49
28	D	27.98		0.17	28.2		-0.35		1.45
29	D	27.22		0.93	27.56		0.29		1.60
30	D	27.63		0.52	27.84		0.01		1.45
31	D	27.23		0.92	27.65		0.20		1.69
32	D	27.79		0.36	28.12		-0.27		1.57
33	Z	28.2		-0.05	28.43		-0.58		1.45
34	Z	27.85		0.30	27.99		-0.14		1.37
35	Z	28.32		-0.17	28.42		-0.57		1.32
36	Z	27.44		0.71	27.52		0.33		1.32
37	Z	28.24		-0.09	28.48		-0.63		1.46
38	Z	28.18		-0.03	28.18		-0.33		1.23
39	Z	27.41		0.74	27.58		0.27		1.41
40	G	27.82		0.33	27.81		0.04		1.23
41	G	28.39		-0.24	28.35		-0.50		1.19
42	G	27.86		0.29	27.97		-0.12		1.34
43	G	27.54		0.61	27.66		0.19		1.36
44	G	27.74		0.41	27.94		-0.09		1.43
45	G	27.56		0.59	27.73		0.12		1.41

	mean valu	standard deviation
K	1.00	0.09
M	2.03	0.20
DX	1.37	0.07
D	1.52	0.11
Z	1.37	0.08
G	1.32	0.08

46 G	27.99	0.16	27.97	−0.12		1.22
47 G	27.91	0.24	28.02	−0.17		1.34

Replicate	Group	NR1<br>Ct (dRn)	Ct_{NR1}正常	ΔCt_{NR1}	β-actin<br>Ct (dRn)	$Ct_{\beta\text{-actin}}$正常	$\Delta Ct_{\beta\text{-actin}}$	Efficiency	Relative Expression Ratio
1	K	28.1	28.47	0.37	27.56	27.85	0.29	$E_{\beta\text{-actin}}$	**1.06**
2	K	28.73		-0.26	28.01		-0.16	103.5%	**0.93**
3	K	28.21		0.26	27.67		0.18	E_{NR1}	**1.06**
4	K	28.94		-0.47	28.16		-0.31	104.7%	**0.89**
5	K	28.64		-0.17	28.04		-0.19		**1.02**
6	K	28.57		-0.10	27.95		-0.10		**1.00**
7	K	28.47		0.00	27.88		-0.03		**1.02**
8	K	28.13		0.34	27.54		0.31		**1.03**
9	M	27.31		1.16	27.91		-0.06		**2.40**
10	M	27.39		1.08	27.92		-0.07		**2.28**
11	M	27.17		1.30	27.53		0.32		**2.03**
12	M	27.79		0.68	28.21		-0.36		**2.11**
13	M	28.03		0.44	28.48		-0.63		**2.15**
14	M	27.9		0.57	28.25		-0.40		**2.00**
15	M	26.87		1.60	27.52		0.33		**2.49**
16	M	28.03		0.44	28.4		-0.55		**2.03**
17	DX	28.55		-0.08	28.46		-0.61		**1.46**
18	DX	28.46		0.01	28.34		-0.49		**1.43**
19	DX	28.28		0.19	28.46		-0.61		**1.77**
20	DX	28.25		0.22	28.13		-0.28		**1.43**
21	DX	27.96		0.51	28.1		-0.25		**1.72**
22	DX	27.57		0.90	27.77		0.08		**1.80**
23	DX	28.06		0.41	28.18		-0.33		**1.70**
24	DX	28		0.47	27.9		-0.05		**1.45**
25	D	28.24		0.23	28.08		-0.23		**1.39**
26	D	28.35		0.12	28.17		-0.32		**1.37**
27	D	28.33		0.14	28.32		-0.47		**1.55**
28	D	28.47		0.00	28.2		-0.35		**1.28**
29	D	27.62		0.85	27.56		0.29		**1.50**
30	D	27.86		0.61	27.84		0.01		**1.54**
31	D	27.97		0.50	27.65		0.20		**1.24**
32	D	28.19		0.28	28.12		-0.27		**1.48**
33	Z	28.82		-0.35	28.43		-0.58		**1.18**
34	Z	28.15		0.32	27.99		-0.14		**1.39**
35	Z	28.6		-0.13	28.42		-0.57		**1.37**
36	Z	28.03		0.44	27.52		0.33		**1.09**
37	Z	28.87		-0.40	28.48		-0.63		**1.18**
38	Z	28.63		-0.16	28.18		-0.33		**1.13**
39	Z	27.8		0.67	27.58		0.27		**1.34**
40	G	28.25		0.22	27.81		0.04		**1.14**
41	G	28.83		-0.36	28.35		-0.50		**1.10**
42	G	28.44		0.03	27.97		-0.12		**1.11**
43	G	27.9		0.57	27.66		0.19		**1.32**
44	G	28.27		0.20	27.94		-0.09		**1.23**
45	G	28.04		0.43	27.73		0.12		**1.25**

	mean valu	standard deviation
K	1.00	0.06
M	2.19	0.17
DX	1.60	0.16
D	1.42	0.11
Z	1.24	0.12
G	1.21	0.08

46 G	28.23	0.24	27.97	−0.12		1.30
47 G	28.38	0.09	28.02	−0.17		1.21
